# Genome-wide analysis of primary CD4+ and CD8+ T cell transcriptomes shows evidence for a network of enriched pathways associated with HIV disease

**DOI:** 10.1186/1742-4690-8-18

**Published:** 2011-03-16

**Authors:** Jing Qin Wu, Dominic E Dwyer, Wayne B Dyer, Yee Hwa Yang, Bin Wang, Nitin K Saksena

**Affiliations:** 1 Retroviral Genetics Division, Center for Virus Research, Westmead Millennium Institute, University of Sydney, Darcy Road, Westmead, NSW 2145, Australia; 2 Department of Virology, Centre for Infectious Diseases and Microbiology Laboratory Services, ICPMR, Westmead Hospital, Westmead, NSW 2145, Australia; 3 Immunovirology Laboratory, Australian Red Cross Blood Service, Sydney, NSW 2000, Australia; 4 School of Mathematics and Statistics, University of Sydney, NSW 2006, Australia

## Abstract

**Background:**

HIV preferentially infects CD4+ T cells, and the functional impairment and numerical decline of CD4+ and CD8+ T cells characterize HIV disease. The numerical decline of CD4+ and CD8+ T cells affects the optimal ratio between the two cell types necessary for immune regulation. Therefore, this work aimed to define the genomic basis of HIV interactions with the cellular transcriptome of both CD4+ and CD8+ T cells.

**Results:**

Genome-wide transcriptomes of primary CD4+ and CD8+ T cells from HIV+ patients were analyzed at different stages of HIV disease using Illumina microarray. For each cell subset, pairwise comparisons were performed and differentially expressed (DE) genes were identified (fold change >2 and B-statistic >0) followed by quantitative PCR validation. Gene ontology (GO) analysis of DE genes revealed enriched categories of complement activation, actin filament, proteasome core and proton-transporting ATPase complex. By gene set enrichment analysis (GSEA), a network of enriched pathways functionally connected by mitochondria was identified in both T cell subsets as a transcriptional signature of HIV disease progression. These pathways ranged from metabolism and energy production (TCA cycle and OXPHOS) to mitochondria meditated cell apoptosis and cell cycle dysregulation. The most unique and significant feature of our work was that the non-progressing status in HIV+ long-term non-progressors was associated with MAPK, WNT, and AKT pathways contributing to cell survival and anti-viral responses.

**Conclusions:**

These data offer new comparative insights into HIV disease progression from the aspect of HIV-host interactions at the transcriptomic level, which will facilitate the understanding of the genetic basis of transcriptomic interaction of HIV *in vivo *and how HIV subverts the human gene machinery at the individual cell type level.

## Background

HIV preferentially infects CD4+ T cells and the functional impairment and numerical decline of CD4+ and CD8+ T cells characterize HIV disease. The numerical decline of CD4+ and CD8+ T cells affects the optimal ratio between the two cell types necessary for immune regulation. This ratio can predict the progression or non-progression to HIV disease [1]. In HIV+ non-progressing individuals, who control viremia in the absence of antiviral therapy, polyclonal, persistent, and vigorous HIV-1-specific CD4+ T cell proliferative responses are present, resulting in the elaboration of interferon and antiviral chemokines [2]. HIV disease progression leads to a wide range of defects in CD4+ T cell function, such as altered profiles of cytokine production [3], weak or absent HIV-specific CD4+ T cell proliferation [4,5], dysregulation of CD4+ T cell turnover [6], and impaired production of new cells [7,8]. The cytotoxic and non-cytotoxic antiviral arms of CD8+ T cells are potent in controlling HIV replication [9]. The non-cytotoxic activity including chemokines, soluble CD8 antiviral factor, urokinase-type plasminogen activator, and antiviral membrane-bound factor suppresses HIV transcription in an antigen-independent and major histocompatibility complex-unrestricted manner [10]. The induction of memory cytotoxic CD8+ T cells in early HIV infection, particularly Gag-specific cells, helps control viral replication and is associated with slower CD4+ T cell decline [11]. Host cytolytic effector responses appear to delay the disease progression [12]. In HIV disease progression, numerical decline and functional impairment of CD8+ T cells can be attributed to increased susceptibility to apoptosis from alterations in the cytokine milieu in lymphoid tissue, bystander effects from neighboring productively infected CD4+ T cells, and toxicity from the release of HIV-derived gp120 or Tat proteins, in addition to direct infection [13,14]. Although the direct and indirect HIV-induced mechanisms leading to CD4+ and CD8+ T cell depletion are known, the genetic basis of these pathogenic mechanisms are uncertain. To better understand HIV pathogenesis at the genomic level, investigators have carried out microarray-based studies of HIV infection, including the use of whole PBMC, cell lines, monocytes, macrophages, T cells, lymphoid and gut tissue [15]. For CD4+ T cells, reports mainly focused on T cell lines *in vitro*, except for one study reporting resting CD4+ T cells in viremic versus aviremic HIV+ individuals [16]. The limitation of *in vitro *studies is that they do not reflect effects observed *in vivo*, as HIV induces T cell dysfunction systemically and affects both the HIV-infected cells and the majority of bystander cells. Studies on CD8+ T cells are limited, and include searching for genes responsible for non-cytotoxic CD8+ T cell activity and comparisons between individuals with high non-cytotoxic activity and uninfected controls [17,18]. Recently, the transcriptional profiling of CD4+ and CD8+ T cells from early infection, chronic infection, and LTNP patients has been reported [19]. Interferon responses as a transcriptional signature of T cells from early and chronically infected patients were identified, but no pronounced difference between early and chronically infected patients, between HIV seronegative controls and LTNPs was detected; thus, combined groups had to be used to facilitate further analysis [19].

Using Illumina Human-6 V2 Expression BeadChips encompassing all 27,000 human genes (=48,000 gene transcripts), recently we have successfully identified coordinated up-regulation of oxidative phosphorylation (OXPHOS) genes as a transcriptional signature in CD8+ T cells from the viremic patients on HAART and the possible association between components of MAPK pathway and LTNP status [20]. Further study suggested a correlation between HIV load level and CD8+ T cell transcriptome shift [21], supporting that detection threshold of viral load could be used as an accurate grouping criteria in differentiating HIV disease status. Here, in this study we compared global gene expression profiles of all 25,000 human genes for both primary CD4+ and CD8+ T cells from three HIV+ disease groups along with healthy HIV seronegative controls. The various HIV+ disease groups included long-term non-progressors (LTNPs) and viremic patients on HAART (VIR), as well as aviremic patients on HAART (below detectable levels, BDL). Using Illumina Human-6 V2 Expression BeadChips, comparative genome-wide transcriptomic analysis of *ex-vivo *collected CD4+ and CD8+ T cells clearly showed evidence for concerted up-regulation of metabolic pathways during HIV disease progression, and a clear correlation between transcriptome shift and detectable plasma viremia uniquely for CD8+ T cells. A novel observation was that HIV non-progression was associated with enriched MAPK, WNT, and AKT pathways. Although both CD4+ and CD8+ T cell transcriptomes showed overlaps at the pathway level, other pathways that segregated these cellular transcriptomes during disease progression were identified, suggesting that HIV also maintains distinct interaction with these cell types *in vivo*. Detection of such transcriptomic signatures for progressive and non-progressive HIV disease may not only facilitate the understanding of genetic basis of HIV interaction with variety of blood leukocytes but also lead to the development of new biomarkers in predicting disease rates.

## Results

### Analysis of differentially expressed genes and enriched gene ontology category

CD4+ and CD8+ T cell-derived total cellular RNA from 14 HIV-infected individuals (4 LTNP, 5 BDL and 5 VIR, Table 1) and 5 HIV seronegative (NEG) healthy individuals were hybridized to the Sentrix Human-6 V2 Expression BeadChip (Singapore). After passing quality assessment, data normalization was performed and a linear model fit in conjunction with an empirical Bayes statistics were used to identify candidate DE genes [22,23]. For both CD4+ and CD8+ T cells, pairwise comparisons from the four study groups (BDL versus NEG, VIR versus NEG, LTNP versus NEG, BDL versus LTNP, VIR versus LTNP, BDL versus VIR) were carried out and candidate DE genes with >2-fold change and B-statistic > 0 were identified for each comparison. The number of DE genes identified in each comparison is listed in Table 2 and the list of DE genes for each comparison between HIV+ disease groups are provided in Additional File 1.

**Table 1:** Patient clinical detail

Patient	Group	Age	Viral load (copies/ml)	CD4 counts (cells/μl)	CD8 counts (cells/μl)
V1	VIR	46	209	300	566

V2	VIR	41	2530	278	845

V3	VIR	40	5710	324	1169

V4	VIR	43	546,000	94	312

V5	VIR	60	683,000	48	73

B1	BDL	48	< 50	450	288

B2	BDL	63	< 50	480	360

B3	BDL	40	< 50	1065	1065

B4	BDL	62	< 50	776	1692

B5	BDL	59	< 50	251	548

L1	LTNP	59	< 50	630	579

L2	LTNP	51	< 50	714	476

L3	LTNP	79	< 50	920	900

L4	LTNP	33	57	780	900

V1-5: viremic patients on HAART; B1-5: aviremic patients on HAART; L1-4: long-term non-progressors. All the patients and seronegative controls are males.

Plasma viral load was measured using the Quantiplex HIV RNA3.0 (Chiron bDNA) assay with a lower limit of detection of 50 HIV-1 copies/ml (Chiron Diagnostics, Halstead, United Kingdom).

**Table 2:** Number of differentially expressed genes in pairwise comparisons for CD4+ and CD8+ T cells (fold change > 2 and B-statistic > 0)

Differentially expressed genes	CD4		CD8		CD4 and CD8	
	
	up	down	up	down	up	down
BDLvsNEG	50	24	206	72	15	3

VIRvsNEG	173	128	477	273	79	48

LTNPvsNEG	5	9	17	6	0	0

BDLvsLTNP	0	3	3	1	0	0

VIRvsLTNP	29	7	118	63	10	2

BDLvsVIR	1	8	5	12	0	1

Up: up-regulation; down: down-regulation; vs: versus; CD4 and CD8: genes differentially expressed in both CD4+ and CD8+ T cells in the same paired comparison.

**Figure 1:**
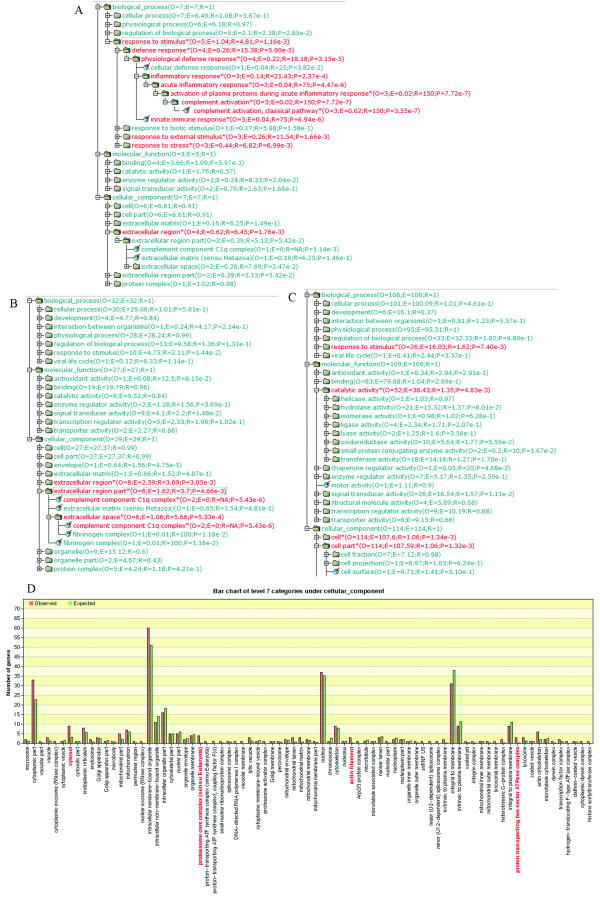
**Gene ontology (GO) tree and bar chart for the enriched GO categories**. GO categories with at least 2 genes and p < 0.01 are identified as enriched and colored red in the GOTree. In GOTree, O stands for observed gene number in the category; E for expected gene number in the category; R for ratio of enrichment for the category; and P for p value calculated from the statistical test given for the categories with R > 1 to indicate the significance of enrichment. A. GO tree for the differentially expressed genes in CD4+ T cells between the BDL and VIR groups. B. GO tree for the differentially expressed genes in CD4+ T cells between the VIR and LTNP groups. C. GO tree for the differentially expressed genes in CD8+ T cells between the VIR and LTNP groups. D. Bar chart of level 7 categories under cellular component category for CD8+ T cells between the VIR and LTNP groups.

To identify the important functional categories from the DE genes, GO Tree was used to identify GO categories with significantly enriched gene numbers (P < 0.01). For BDL versus VIR and VIR versus LTNP comparisons in CD4+ T cells, the GO categories response to stimuli and extracellular region were significantly enriched (p <0.01; Figure 1A and 1B). The sub-tree view under the above categories revealed that both complement activation with contributing genes C1QB, C1QC, and SERPING1, and complement component C1q complex with contributing genes C1QA and C1QB were significantly enriched. For the VIR and LTNP comparison in CD8+ T cells, response to stimuli, catalytic activity, and cell part were significantly enriched (Figure 1C). Further inspection of these enriched categories showed that at level 7, category cytosol with contributing genes BAG3, PRF1, UNC119, ARFIP1, PSME2, PSMA5, PSMB2, PSMB8, and PSMB10, category actin filament with contributing genes IQGAP1, ACTB, and ACTA2, category proteasome core complex with contributing genes PSMA5, PSMB2, PSMB8, and PSMB10, and category proton-transporting ATPase complex with contributing genes ATP5J2, ATP6V0E1, and ATP6V1 D were significantly enriched (Figure 1D).

### Validation of differentially expressed genes

To confirm the DE genes from the Illumina microarray, mRNA expression levels of the selected DE genes from each paired comparison for both CD4+ and CD8+ T cells were measured by quantitative real-time PCR (Table 3). DE genes contributing to the enriched GO categories were randomly selected for real-time PCR confirmation. For CD8+ T cells, these genes included BAG3 in category cytosol, ACTA2 in category actin, PSMB2 and PSMA5 in category proteasome core complex, and ATP6V1 D in category proton-transporting ATPase complex. For CD4+ T cells, C1QB, C1QC, and SERPING1 in category complement activation were selected. DE genes not under any enriched GO categories were also randomly selected. The mRNA from the CD4+ and CD8+ T cells of the same patient at the same time point was used for real-time multiplexed qPCR analysis. The fold changes were evaluated by real-time multiplexed qPCR and were well consistent with the results from differentially expressed genes obtained by microarray (Table 3).

**Table 3:** qPCR validation of differentially expressed genes

Gene Symbol	**Accesion No**.	Description	Fwd Primer	Fwd Primer Seq	Rev Primer	Rev Primer Seq	Paired Comparison	Cell Type	FC qPCR	FC MA
KLRD1	NM_002262.2	killer cell lectin-like receptor subfamily D, member 1	KLRD1L	gtgggagaatggctctgc	KLRD1R	tttgtattaaaagtttcaaatgatgga	BDLvsLTNP	CD8	2.5	2.1

IRS2	NM_003749.2	insulin receptor substrate 2	IRS2L	tgacttcttgtcccaccactt	IRS2R	catcctggtgataaagccaga	BDLvsVIR	CD8	3.8	2.7

GBP1	NM_002053.2	guanylate binding protein 1, interferon-inducible	GBP1L	aggccacatcctagttctgc	GBP1R	tccaggagtcattctggttgt	BDLvsVIR	CD8	-2.5	-2.4

ACTA2	NM_001613.1	actin, alpha 2, smooth muscle, aorta	ACTA2L	ctgttccagccatccttcat	ACTA2R	tcatgatgctgttgtaggtggt	BDLvsVIR	CD8	-1.3	-2.2

ATP6V1D	NM_015994.2	ATPase, H+ transporting, lysosomal 34kDa, V1 subunit D	ATP6V1DL	ttttcactagctgaagccaagtt	ATP6V1DR	gcgctttattgacattttggat	VIRvsLTNP	CD8	2.0	2.8

BAG3	NM_004281.3	BCL2-associated athanogene 3	BAG3L	cagccagataaacagtgtggac	BAG3R	agaggcagctggagactgg	VIRvsLTNP	CD8	-1.5	-2.4

ACTA2	NM_001613.1	actin, alpha 2, smooth muscle, aorta	ACTA2L	ctgttccagccatccttcat	ACTA2R	tcatgatgctgttgtaggtggt	VIRvsLTNP	CD8	4.3	2.8

PSMB2	NM_002794.3	proteasome subunit, beta type, 2	PSMB2L	agagggcagtggaactcctt	PSMB2R	gaaggttggcagattcagga	VIRvsLTNP	CD8	1.3	2.3

PSMA5	NM_002790.2	proteasome subunit, alpha type, 5	PSMA5L	tgaatgcaacaaacattgagc	PSMA5R	ttcttcctttgtgaacatgtgg	VIRvsLTNP	CD8	2.7	2.7

C1QB	NM_000491.3	complement component 1, q subcomponent, B chain	C1QBL	ggcctcacaggacaccag	C1QBR	ccatgggatcttcatcatcata	BDLvsVIR	CD4	-4.8	-4.8

C1QC	NM_172369.3	complement component 1, q subcomponent, C chain	C1QCL	aaggatgggtacgacggact	C1QCR	ttctgccctttgggtcct	BDLvsVIR	CD4	-5.6	-4.1

SERPING1	NM_000062.2	serpin peptidase inhibitor, clade G (C1 inhibitor), member 1,	SERPING1L	ctccttacccaggtcctgct	SERPING1R	ggatgctctccaggtttgtt	BDLvsVIR	CD4	-5.0	-2.6

C1QB	NM_000491.3	complement component 1, q subcomponent, B chain	C1QBL	ggcctcacaggacaccag	C1QBR	ccatgggatcttcatcatcata	VIRvsLTNP	CD4	6.1	6.0

C1QC	NM_172369.3	complement component 1, q subcomponent, C chain	C1QCL	aaggatgggtacgacggact	C1QCR	ttctgccctttgggtcct	VIRvsLTNP	CD4	7.3	4.4

SERPING1	NM_000062.2	serpin peptidase inhibitor, clade G (C1 inhibitor), member 1,	SERPING1L	ctccttacccaggtcctgct	SERPING1R	ggatgctctccaggtttgtt	VIRvsLTNP	CD4	5.3	2.8

FC_qPCR: fold change by qPCR; FC_MA: fold change by microarray

### Gene set enrichment analysis

**Figure 2:**
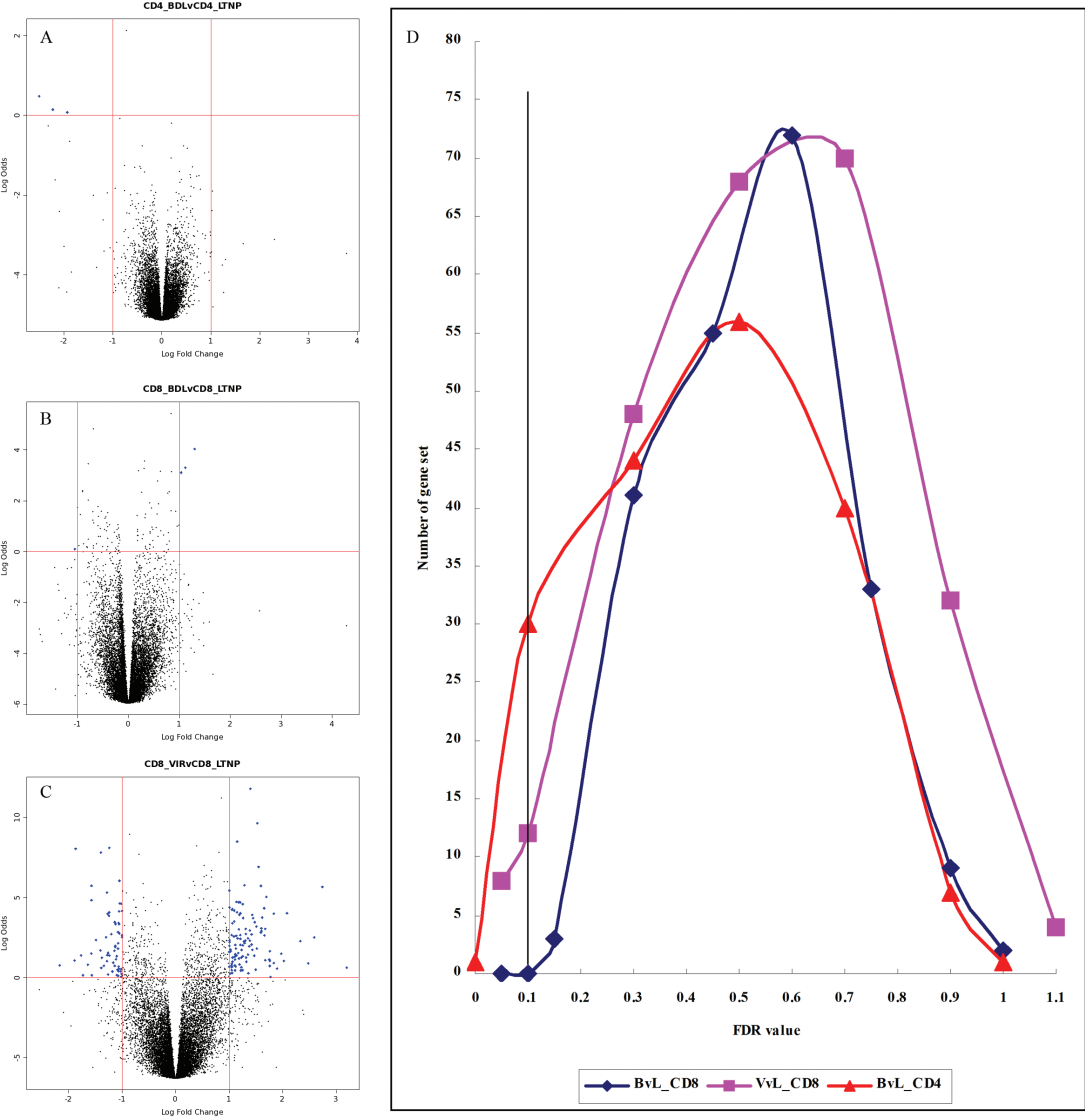
**Gene set number plots against the FDR value from GSEA and the corresponding volcano plots visualizing the number of differentially expressed genes**. Each differentially expressed gene is represented by a blue dot. A. Volcano plot for CD4+ T cells between BDL and LTNP groups. B. Volcano plot for CD8+ T cells between BDL and LTNP groups. C. Volcano plot for CD8+ T cells between VIR and LTNP groups. D. Plot of gene set numbers against FDR value (BDL versus LTNP and VIR versus LTNP in CD8+ T cells, BDL versus LTNP in CD4+ T cells).

To further unravel the biological mechanisms differentiating between HIV disease groups, pairwise comparisons using GSEA were performed for both CD4+ and CD8+ T cells from three HIV+ groups (VIR versus BDL, VIR versus LTNP, and BDL versus LTNP). Rather than single DE genes, GSEA evaluates microarray data at the biological pathway level by performing unbiased global searches for genes that are coordinately regulated in predefined gene sets [24]. The number of significantly enriched gene sets (FDR < 0.05/0.1) in each pairwise comparison is listed in Table 4. The representative plots of gene set numbers against the FDR value (BDL versus LTNP and VIR versus LTNP in CD8+ T cells, BDL versus LTNP in CD4+ T cells) along with the corresponding volcano plots visualizing the number of differentially expressed genes are shown in Figure 2.

**Table 4:** Number of enriched gene sets in pairwise comparisons for CD4+ and CD8+ T cells using gene set enrichment analysis (at level of FDR < 0.05 and FDR < 0.1)

FDR < 0.05	CD4		CD8		CD4 and CD8	
Enriched gene sets	up	down	up	down	up	down

VIRvsBDL	19	3	29	2	5	2

VIRvsLTNP	27	2	8	0	6	0

BDLvsLTNP	20	7	0	1	0	1

**FDR < 0.1**	**CD4**		**CD8**		**CD4 and CD8**	

Enriched gene sets	up	down	up	down	up	down

VIRvsBDL	57	3	53	2	18	2

VIRvsLTNP	51	4	20	0	13	0

BDLvsLTNP	31	34	0	5	0	3

Up: up-regulation; down: down-regulation; vs: versus; CD4 and CD8: gene sets enriched in both CD4+ and CD8+ T cells in the same paired comparison.

#### Metabolic pathways associated with HIV disease progression

In CD4+ and/or CD8+ T cells between HIV+ disease groups, 43 metabolic pathways were significantly up-regulated in the first group in at least one of the above pairwise comparisons when comparing the first (more advanced disease status) to the second group (less advanced disease status) as listed in Table 5. According to the biological function, these 43 pathways were divided into (1) aerobic metabolism; (2) carbohydrate and lipid metabolism; (3) amino acid and nucleotide metabolism; and (4) protein metabolism, respectively. Under each category, the pathways that showed significance across more pairwise comparisons were listed at the top.

**Table 5:** Enriched gene sets involved in energy production

Gene set name	VvsB_CD8	VvsB_CD4	VvL_CD8	VvL_CD4	BvsL_CD4
**Aerobic metabolism**					

HSA00190_OXIDATIVE_PHOSPHORYLATION	0.05	0.05	0.05	0.05	0.05

HSA00020_CITRATE_CYCLE	0.1	0.1	0.1		0.1

HSA00760_NICOTINATE_AND_NICOTINAMIDE_METABOLISM	0.05	0.05	0.1	0.05	

TYPE_III_SECRETION_SYSTEM		0.05		0.05	0.05

PHOTOSYNTHESIS		0.05		0.05	0.05

ATP_SYNTHESIS		0.05		0.05	0.05

FLAGELLAR_ASSEMBLY		0.05		0.05	0.05

MITOCHONDRIAL_FATTY_ACID_BETAOXIDATION	0.1			0.1	

PYRUVATE_METABOLISM			0.1		0.05

**Carbohydrate and lipid metabolism**					

HSA00650_BUTANOATE_METABOLISM	0.05	0.1		0.05	0.05

HSA00071_FATTY_ACID_METABOLISM	0.05			0.05	0.05

HSA00670_ONE_CARBON_POOL_BY_FOLATE	0.1			0.1	0.1

HSA00051_FRUCTOSE_AND_MANNOSE_METABOLISM	0.1	0.1		0.1	

HSA00511_N_GLYCAN_DEGRADATION		0.05		0.05	0.1

HSA00030_PENTOSE_PHOSPHATE_PATHWAY	0.1			0.1	

HSA01032_GLYCAN_STRUCTURES_DEGRADATION		0.05		0.05	

STARCH_AND_SUCROSE_METABOLISM		0.05		0.1	

HSA00052_GALACTOSE_METABOLISM		0.1	0.1		

HSA00532_CHONDROITIN_SULFATE_BIOSYNTHESIS		0.1		0.05	

PROPANOATE_METABOLISM	0.05				

HSA00040_PENTOSE_AND_GLUCURONATE_INTERCONVERSIONS	0.1				

HSA00010_GLYCOLYSIS_AND_GLUCONEOGENESIS	0.1				

GLYCOLYSIS	0.1				

GLUCONEOGENESIS	0.1				

HSA00710_CARBON_FIXATION				0.1	

PROSTAGLANDIN_AND_LEUKOTRIENE_METABOLISM				0.1	

BILE_ACID_BIOSYNTHESIS					0.05

HSA00531_GLYCOSAMINOGLYCAN_DEGRADATION		0.1			

HSA00565_ETHER_LIPID_METABOLISM		0.1			

FRUCTOSE_AND_MANNOSE_METABOLISM		0.1			

**Amino acid and nucleotide metabolism**					

HSA00280_VALINE_LEUCINE_AND_ISOLEUCINE_DEGRADATION	0.05	0.1	0.05	0.05	0.05

LYSINE_DEGRADATION	0.05		0.1	0.05	0.05

HSA00230_PURINE_METABOLISM	0.05	0.1	0.1	0.1	

PHENYLALANINE_METABOLISM		0.1	0.05	0.05	0.1

HSA00380_TRYPTOPHAN_METABOLISM		0.1	0.1	0.05	0.1

HSA00240_PYRIMIDINE_METABOLISM	0.05	0.1		0.05	

HSA00252_ALANINE_AND_ASPARTATE_METABOLISM	0.1			0.1	0.05

HSA00410_BETA_ALANINE_METABOLISM	0.05			0.1	

PORPHYRIN_AND_CHLOROPHYLL_METABOLISM	0.05	0.1		0.05	0.1

HSA00330_ARGININE_AND_PROLINE_METABOLISM				0.1	

**Protein metabolism**					

PROTEASOME	0.05	0.1	0.05	0.05	0.05

PROTEASOMEPATHWAY		0.1		0.1	0.1

HSA00970_AMINOACYL_TRNA_BIOSYNTHESIS	0.05			0.1	

The presence of curious gene groups photosynthesis, Type III secretion and flagellar assembly is due to the heavy overlapping genes between these pathways and OXPHOS pathway we have found significant in our study.  For example, in the group comparison, VIR versus BDL in CD4 T cells, 53 core enrichment genes contributed to the enrichment of OXPHOS pathway and 10 core enrichment genes contributed to the flagellar assembly pathway. All these 10 core genes from flagellar assembly pathway ATP6V1D, ATP6V0D1, ATP6V1A, ATP6V0A1, ATP6V1B2, ATP6AP1, ATP6V1E1, ATP6V1H, ATP6V0B, and ATP6V1F are ALL present in the 53 core genes from OXPHOS pathway. In this overlapping situation, when you do Gene Set Enrichment Analysis (GSEA) using the gene set file (gmt file constructed by gene symbols downloaded from broad institute website), the flagellar pathway will appear together with OXPHOS pathway as the same gene symbol applies to different species and these 2 pathways do have a number of overlapping genes. It is common that at the level of pathway analysis, pathways with certain amount of overlapping genes will always appear together, just like flagellar assembly showed up with the OXPHOS. Our results just reflected the true output from GSEA analysis. We leave them there but at the bottom of the aerobic metabolism category with the consideration that it reflects the same biological theme related to ATP production even though from different species.

VvsB_CD8: Gene sets enriched in the VIR group in the comparison of VIR versus BDL in CD8+ T cells

VvsB_CD4: Gene sets enriched in the VIR group in the comparison of VIR versus BDL in CD4+ T cells

VvL_CD8: Gene sets enriched in the VIR group in the comparison of VIR versus LTNP in CD8+ T cells

VvL_CD4: Gene sets enriched in the VIR group in the comparison of VIR versus LTNP in CD4+ T cells

BvsL_CD4: Gene sets enriched in the BDL group in the comparison of BDL versus LTNP in CD4+ T cells

Gene sets significantly enriched are marked by the number 0.05 or 0.1 in the corresponding paired comparisons 0.05: FDR < or = 0.05; 0.1: FDR < or = 0.1

Gene set information could be searched at the website http://www.broad.mit.edu/gsea/msigdb/search.jsp

**Figure 3:**
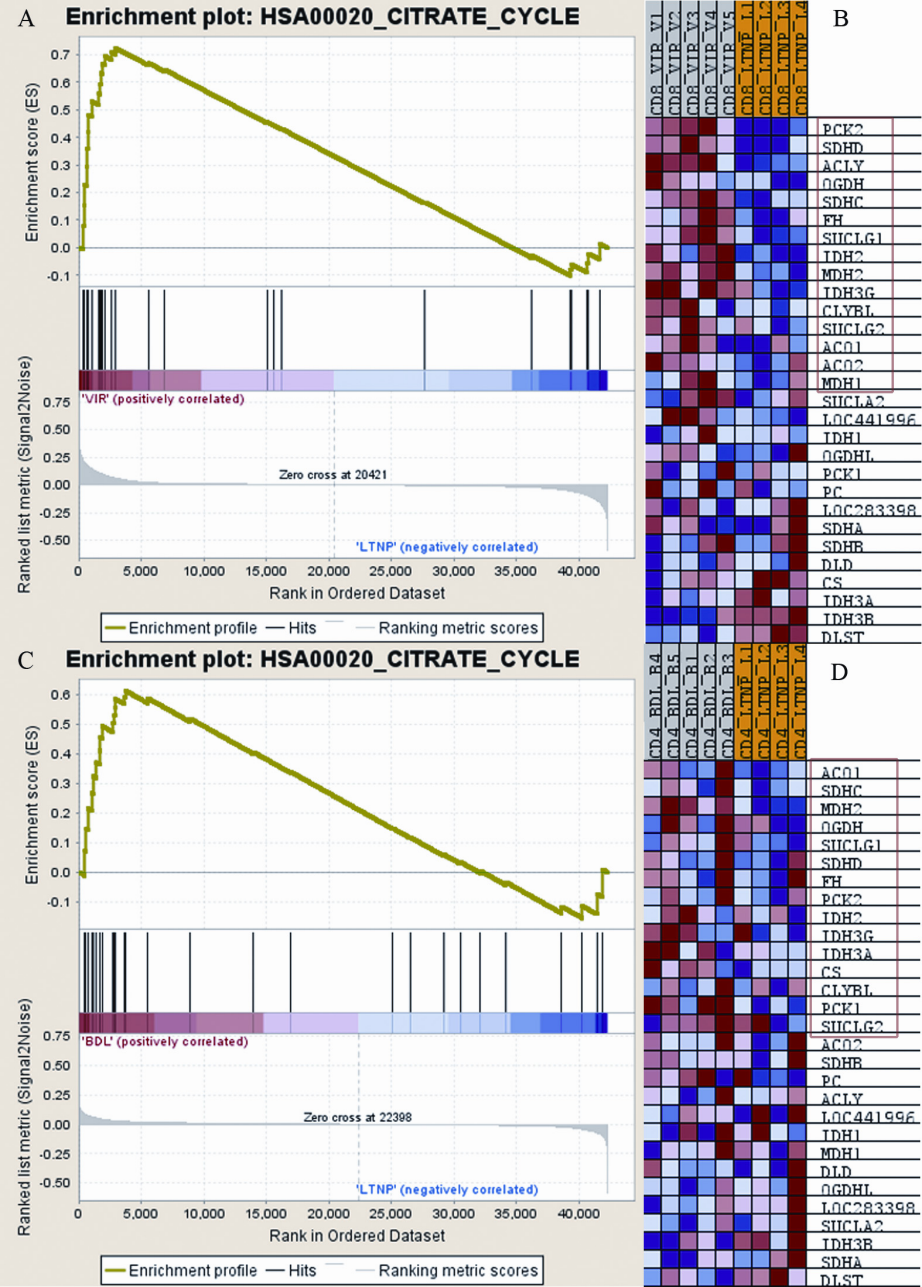
**Enrichment plot and heat map for the gene set of tricarboxylic acid cycle by GSEA**. A. Enrichment plot for CD8+ T cells from the VIR group (VIR versus LTNP). Bottom, plot of the ranked list of all genes. Y axis, value of the ranking metric; X axis, the rank for all genes. Genes whose expression levels are most closely associated with the VIR or LTNP group get the highest metric scores with positive or negative sign, and are located at the left or right edge of the list. Middle, the location of genes from the gene set TCA cycle within the ranked list. Top, the running enrichment score for the gene set as the analysis walks along the ranked list. The score at the peak of the plot is the enrichment score (ES) for this gene set and those genes appear before or at the peak are defined as core enrichment genes for this gene set. B. Heat map of the genes within the gene set of TCA cycle corresponding to A. The genes that contribute most to the ES, i.e., genes that appear in the ranked list before or at the peak point of ES, are defined as core enrichment genes and highlighted by the red rectangle. Rows, genes, columns, samples. Range of colors (red to blue) shows the range of expression values (high to low). C. Enrichment plot for CD4+ T cells from the BDL group (BDL versus LTNP). D. Heat map of the genes within the gene set of TCA cycle corresponding to C.

**Figure 4:**
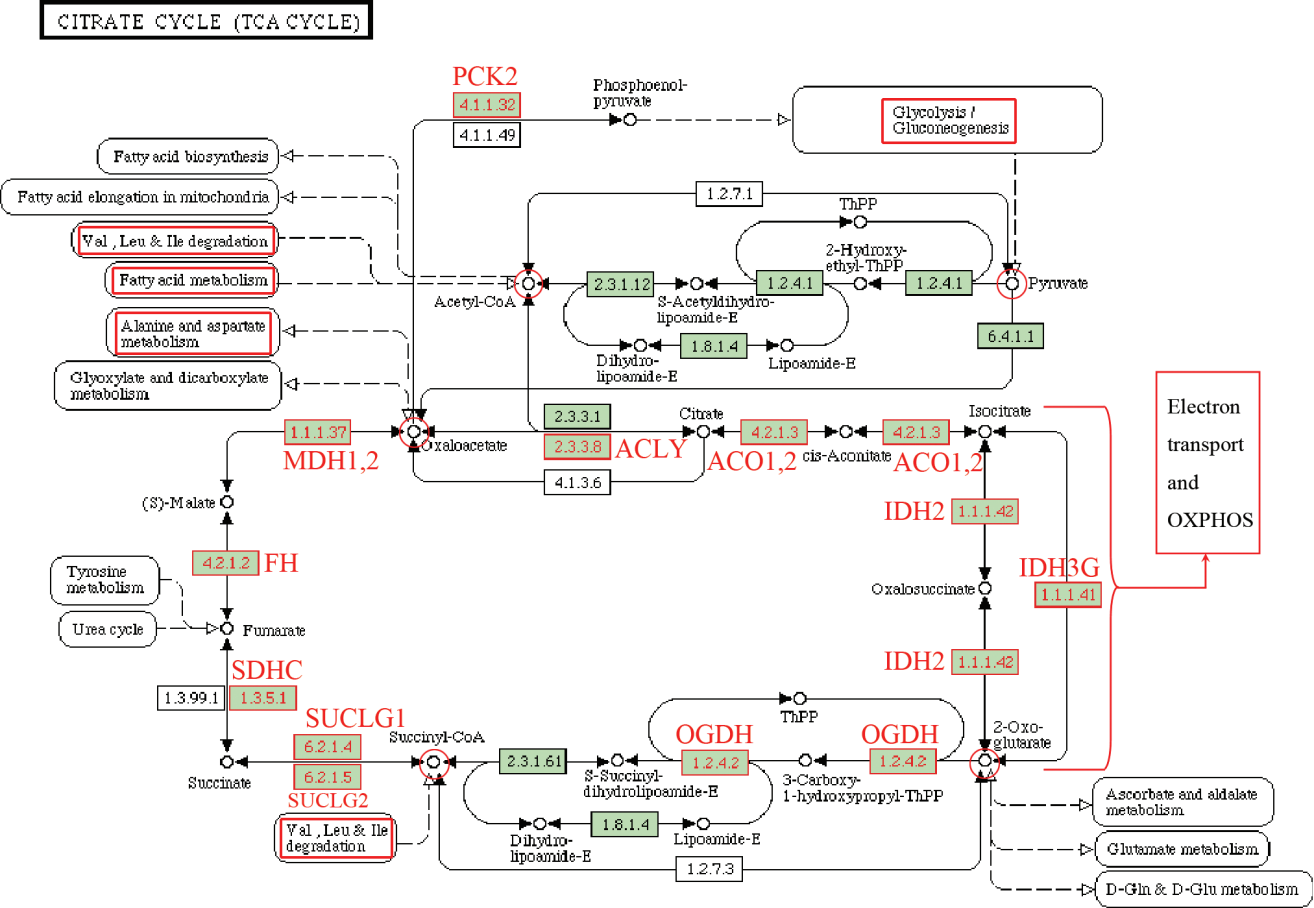
**Coordinately up-regulated TCA cycle genes in CD8+ T cells from the VIR group (VIR versus LTNP) illustrated in TCA cycle pathway from Kyoto Encyclopedia of Genes and Genomes (KEGG; **http://www.genome.jp/kegg/**)**. The enzymes encoded by coordinately up-regulated TCA cycle genes are highlighted in red and these include ATP citrate lyase (EC:2.3.3.8; gene symbol ACLY), aconitase 1, soluble and aconitase 2, mitochondrial (EC:4.2.1.3; gene symbol ACO1 and ACO2), isocitrate dehydrogenase 2 (NADP+), mitochondrial (EC:1.1.1.42; gene symbol IDH2), isocitrate dehydrogenase 3 (NAD+) gamma (EC:1.1.1.41; gene symbol IDH3G), oxoglutarate (alpha-ketoglutarate) dehydrogenase (EC:1.2.4.2; gene symbol OGDH), succinate-CoA ligase, alpha subunit (EC:6.2.1.4; gene symbol SUCLG1), succinate-CoA ligase, ADP-forming, beta subunit (EC:6.2.1.5; gene symbol SUCLG2), succinate dehydrogenase complex, subunit C (EC:1.3.5.1; gene symbol SDHC), fumarate hydratase (EC:4.2.1.2; gene symbol FH), malate dehydrogenase 1 and 2, (EC:1.1.1.37; gene symbol MDH1 and MDH2), and phosphoenolpyruvate carboxykinase 2 (mitochondrial) (EC:4.1.1.32; gene symbol PCK2). Coordinately up-regulated pathways, which are closely articulated with the TCA cycle, are highlighted by red rectangles. The TCA cycle intermediates linked to other pathways are highlighted by red circles.

In aerobic metabolism, the most generally up-regulated pathways were tricarboxylic acid (TCA) cycle and OXPHOS, central for cell energy production. The OXPHOS pathway was enriched in 5/6 paired comparisons with FDR < 0.05, which reached the most stringent statistical level. Closely associated with OXPHOS pathway is the TCA cycle, which produces immediate precursor (NADH) to OXPHOS to produce ATP. The TCA cycle was up-regulated in 4/6 paired comparisons at the significance level of FDR < 0.1 (FDR cut off value, normally <0.25, more stringently <0.1, most stringently <0.05). To illustrate the up-regulation of TCA cycle in GSEA output, the enrichment plot and heat map of the genes involved in this pathway from the paired comparisons VIR versus LTNP in CD8+ T cells and BDL versus LTNP in CD4+ T cells were shown as representatives in Figure 3. Figure 3B in particular showed that all the patients in the VIR group had consistent up-regulation of TCA cycle genes when compared to the LTNP group irrespective of the range of the viral load. Within the VIR group, V4 and V5, with higher viral load, had even higher expression than V1-V3, with lower viral load. To demonstrate the location of the coordinately up-regulated genes in the TCA cycle, the core enrichment genes closely associated with the VIR group (versus LTNP) in CD8+ T cells are shown as a representative in Figure 4; the close linkages between TCA cycle and other metabolic pathways including OXPHOS, carbohydrates, lipid, and amino acid metabolisms are also illustrated.

In the remaining three categories, butanoate and fatty acid metabolism were top listed in carbohydrate and lipid metabolism. The valine, leucine, and isoleucine degradation in nitrogen metabolism and the proteasome involved in protein degradation were the two most significant and generally enriched pathways besides the OXPHOS pathway (FDR < 0.5 in four paired comparisons and FDR < 0.1 in one paired comparison).

#### Immune-related pathways associated with HIV disease progression

In addition to the metabolic pathways, 39 immune-related gene sets were found to be significantly up-regulated in at least one of the above pairwise comparisons (Table 6; pathways showing significance across more pairwise comparisons are listed at top). Four outstanding groups emerged based on the similarity of biological relevance of these pathways: (1) cell cycle and apoptosis related; (2) cytotoxicity, complement activation, and cell signaling; (3) interleukin and interferon responses; and (4) cytoskeleton and cell adhesion.

**Table 6:** Enriched gene sets other than involved in energy production

Gene set name	VvsB_CD8	VvsB_CD4	VvL_CD8	VvL_CD4	BvsL_CD4
**Cell cycle and apoptosis related**					

CHEMICALPATHWAY	0.1		0.1		

APOPTOSIS		0.1		0.1	

APOPTOSIS_GENMAPP		0.1			

CASPASEPATHWAY				0.1	0.05

SA_CASPASE_CASCADE					0.05

DNA_REPLICATION_REACTOME	0.05		0.05		0.1

G1_TO_S_CELL_CYCLE_REACTOME	0.05				

HSA04110_CELL_CYCLE	0.05				

CELL_CYCLE_KEGG	0.05				

CELLCYCLEPATHWAY	0.05				

G2PATHWAY	0.1				

G1PATHWAY	0.1				

P53PATHWAY	0.1				

MPRPATHWAY	0.1				

ST_GA12_PATHWAY		0.05			

**Cytotoxicity, complement activation and cell signalling**					

HSA04612_ANTIGEN_PROCESSING_AND_PRESENTATION	0.05	0.05	0.05		

HSA04650_NATURAL_KILLER_CELL_MEDIATED_CYTOTOXICITY	0.05	0.05		0.05	

COMPPATHWAY		0.05		0.05	0.05

HSA04610_Complement_and_coagulation-cascades		0.05		0.05	

INTRINSICPATHWAY		0.1		0.1	

HSA04620_TOLL_LIKE_RECEPTOR_SIGNALING_PATHWAY		0.05		0.1	

TOLLPATHWAY		0.05			

HSA04662_B_CELL_RECEPTOR_SIGNALING_PATHWAY		0.1			

SA_B_CELL_RECEPTOR_COMPLEXES		0.1			

**Cytoskeleton and cell adhesion**					

RHOPATHWAY	0.05		0.05	0.05	0.05

NDKDYNAMINPATHWAY	0.1				

SIG_REGULATION_OF_THE_ACTIN_CYTOSKELETON_BY_RHO_GTPASES			0.1		

INTEGRIN_MEDIATED_CELL_ADHESION_KEGG		0.1			

**Interleukin**					

IL12PATHWAY	0.1				

NO2IL12PATHWAY	0.1				

TIDPATHWAY		0.1			

IL6PATHWAY		0.1			

IL3PATHWAY		0.1			

**Other**					

SPPAPATHWAY		0.1			

HSA05120_EPITHELIAL_CELL_SIGNALING_IN_HELICOBACTER_PYLORI_INFECTION		0.05			

HSA05131_PATHOGENIC_ESCHERICHIA_COLI_INFECTION_EPEC		0.1			

HSA05130_PATHOGENIC_ESCHERICHIA_COLI_INFECTION_EHEC		0.1			

HSA04320_DORSO_VENTRAL_AXIS_FORMATION		0.1			

HSA05219_BLADDER_CANCER		0.1			

VvsB_CD8: Gene sets enriched in the VIR group in the comparison of VIR versus BDL in CD8+ T cells

VvsB_CD4: Gene sets enriched in the VIR group in the comparison of VIR versus BDL in CD4+ T cells

VvL_CD8: Gene sets enriched in the VIR group in the comparison of VIR versus LTNP in CD8+ T cells

VvL_CD4: Gene sets enriched in the VIR group in the comparison of VIR versus LTNP in CD4+ T cells

BvsL_CD4: Gene sets enriched in the BDL group in the comparison of BDL versus LTNP in CD4+ T cells

Gene sets significantly enriched are marked by the number 0.05 or 0.1 in the corresponding paired comparisons 0.05: FDR < or = 0.05; 0.1: FDR < or = 0.1

Gene set information could be searched at the website http://www.broad.mit.edu/gsea/msigdb/search.jsp

In the cell cycle and apoptosis category, five pathways were directly involved in cell apoptosis including chemical pathway, apoptosis, apoptosis_genmapp, caspase pathway, and SA_caspase_cascade (Table 6). In CD8+ T cells, the chemical pathway was significantly enriched (FDR < 0.1) when comparing the VIR group against the BDL/LTNP groups. In the comparison between VIR and BDL, 15/21 genes in this pathway were core enrichment genes associated with the VIR group, including STAT1, BCL2L1, CASP7, TLN1, EIF2S1, BCL2, APAF1, BID, BAX, CASP6, PXN, CASP3, PRKCB1, TP53, and AKT1 (Additional File 2). In CD4+ T cells, the apoptosis pathway was significantly enriched (FDR < 0.1) when comparing the VIR group with the BDL/LTNP groups. In comparing the VIR and LTNP groups, 25/66 genes in this pathway were the core enrichment genes associated with the VIR group, such as the death receptor TNFR1 (tumor necrosis factor receptor 1), cytoplasmic adaptor TRADD, RIP1, and TRAF2, cytoplasmic effector DFF45 and DFF40, effector caspase7, and mitochondrial function genes such as BID and BCL2 (Additional File 2).

**Figure 5:**
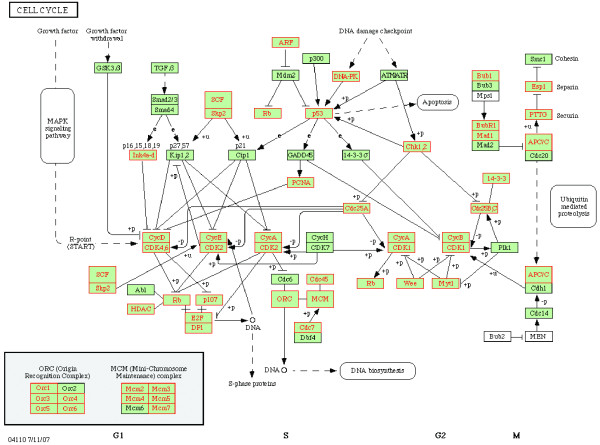
**Coordinately up-regulated cell cycle genes in CD8+ T cells from the VIR group (VIR versus BDL) illustrated in cell cycle pathway from Kyoto Encyclopedia of Genes and Genomes (KEGG; **http://www.genome.jp/kegg/**)**. The proteins encoded by coordinately up-regulated cell cycle genes are highlighted in red.

In relation to cell cycle, six pathways were significantly up-regulated (four with FDR < 0.05 and two with FDR < 0.1) in CD8+ T cells in the VIR group (versus BDL; Table 6). Further inspection of the HSA04110 cell cycle pathway revealed that 54/112 genes were core enrichment genes and the coordinated up-regulation of these genes appears to promote G1 to S transition and induce arrest in G2 to M transition (Figure 5). Coordinately up-regulated genes encoding for proteins promoting G1 to S transition include (1) 2 cyclin dependent kinase (CDK)-cyclin complexes, CDK4/6-cyclin D and CDK2-cyclin E; (2) 2 transcription factors E2F and DP1; (3) DNA biosynthesis complex ORC (origin recognition complex); (4) mini-chromosome maintenance (MCM) complex; (5) CDC25A; and (6) S-phase kinase-associated protein 1 and 2 (SCF and SKP2). Although a few up-regulated genes inhibiting the transition were also present such as INK4a-d and PCNA, the up-regulation of DNA biosynthesis complexes suggested directly promoted transition. Associated with G2 to M transition, we observed up-regulated genes encoding for proteins that generally prevent the cell cycle, including (1) the protein kinases WEE1 and MYT1, which inactivate the complex CDK1-cyclin B pivotal in regulating G2 to M transition; (2) protein 14-3-3 which inactivates CDC25 phosphatase required for CDK1 activation; (3) DNA-PK activated by DNA damage and CHK kinases which inactivates CDC25; (4) p53 which turns on the expression of GADD45 and 14-3-3σ, both prevent the activity of CDK1-cyclin B.

In the category of cytotoxicity, complement activation and cell signaling, pathways of antigen processing and presentation and cell cytotoxicity were significantly enriched in both CD4+ and CD8+ T cells when the VIR group was compared to the BDL group (FDR < 0.05; Table 6). Three complement-associated pathways COMPPATHWAY, HSA04610 complement and coagulation-cascades, and intrinsic pathway as well as toll receptor signaling pathway were significantly and uniquely enriched in CD4+ T cells of the VIR group (versus BDL/LTNP; FDR < 0.05/0.1; Table 6). Further inspection of HSA04610 pathway revealed 18/68 genes were core enrichment genes (Additional File 3).

In the cytoskeleton and cell adhesion category, the RHO pathway was significantly enriched in both CD4+ and CD8+ T cells in the more advanced disease group in four paired comparisons with FDR < 0.05 (Table 6). This pathway is involved in cytoskeleton reorganization, reported to enhance virus fusion to host cell membranes [25]. In the category of interleukin and interferon responses, IL3, IL6, and IL12 pathways were found to be enriched in either CD4+ or CD8+ T cells when the VIR group was compared to the BDL group. For the same paired comparison, the TIDPATHWAY involved in interferon-γ stimulating anti-viral responses was enriched in CD4+ T cells from the VIR group.

Relatively few pathways were significantly up-regulated in the second group (BDL or LTNP) in pairwise comparisons of the VIR versus BDL, VIR versus LTNP, and BDL versus LTNP groups. However, it was noted that the comparison of BDL versus LTNP in CD4+ T cells gave 7 and 27 pathways enriched in the LTNP group at the statistical level of FDR < 0.05 and FDR < 0.1, respectively (Table 7). Out of these 34 gene sets, 15 were closely associated with the MAPK pathway and 10 with cell signaling such as TCR and chemokine and cytokine pathways.

**Table 7:** Enriched pathways in CD4+ T cells from the LTNP group (BDL versus LTNP)

Gene set name	Gene set size	NES	NOM p-val	FDR
**MAPK pathway associated**				

NTHIPATHWAY	22	-2.05	0.000	0.008

ST_JNK_MAPK_PATHWAY	40	-1.88	0.002	0.038

ST_GRANULE_CELL_SURVIVAL_PATHWAY	26	-1.77	0.002	0.049

IGF1PATHWAY	20	-1.77	0.002	0.049

INSULINPATHWAY	21	-1.79	0.004	0.050

NGFPATHWAY	19	-1.81	0.002	0.053

CARDIACEGFPATHWAY	17	-1.75	0.000	0.056

41BBPATHWAY	18	-1.74	0.014	0.058

CDMACPATHWAY	16	-1.71	0.010	0.073

HSA04012_ERBB_SIGNALING_PATHWAY	87	-1.70	0.008	0.074

SA_TRKA_RECEPTOR	16	-1.70	0.012	0.075

HSA04010_MAPK_SIGNALING_PATHWAY	256	-1.64	0.000	0.087

PDGFPATHWAY	27	-1.66	0.012	0.088

TPOPATHWAY	23	-1.66	0.012	0.089

EGFPATHWAY	27	-1.66	0.018	0.091

**Cell signaling**				

INFLAMPATHWAY	29	-1.99	0.000	0.017

CYTOKINEPATHWAY	20	-1.95	0.000	0.023

CCR5PATHWAY	18	-1.78	0.006	0.049

TCRPATHWAY	43	-1.80	0.006	0.052

IL6PATHWAY	21	-1.79	0.002	0.054

IL1RPATHWAY	32	-1.82	0.000	0.055

IL12PATHWAY	20	-1.68	0.018	0.083

TOLLPATHWAY	34	-1.66	0.012	0.086

HSA04060_CYTOKINE_CYTOKINE_RECEPTOR_INTERACTION	253	-1.65	0.000	0.087

HSA04660_T_CELL_RECEPTOR_SIGNALING_PATHWAY	93	-1.65	0.006	0.089

CALCINEURIN_NF_AT_SIGNALING	92	-1.92	0.000	0.030

**Other**				

HYPERTROPHY_MODEL	17	-1.91	0.000	0.025

ATMPATHWAY	19	-1.86	0.000	0.043

HSA05210_COLORECTAL_CANCER	85	-1.78	0.000	0.049

SMOOTH_MUSCLE_CONTRACTION	140	-1.83	0.000	0.051

CIRCADIAN_EXERCISE	40	-1.79	0.000	0.051

GPCRPATHWAY	35	-1.76	0.006	0.054

ST_DIFFERENTIATION_PATHWAY_IN_PC12_CELLS	42	-1.73	0.002	0.064

P53HYPOXIAPATHWAY	19	-1.68	0.022	0.082

Gene set size: number of genes in a particular gene set; NES: normalized enrichment score;

NOM p-val: nominal p value; FDR: false discovery rate

Gene set information could be searched at the website http://www.broad.mit.edu/gsea/msigdb/search.jsp

#### Unique pathways associated with non-progressive HIV disease

**Figure 6:**
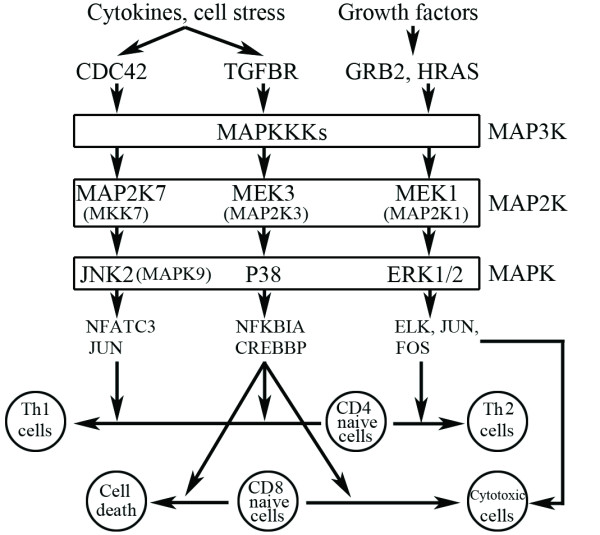
**Branches of MAPK pathway significantly enriched in CD4+ T cells from the LTNP group (BDL versus LTNP)**. Listed genes except for p38 and ERK1/2 are the core enrichment genes derived from MAPK associated pathways and directly involved in the MAPK cascade. The possible roles of each branch of MAPK pathway contributing to the LTNP status are also shown.

Among the 15 MAPK-associated pathways significantly enriched in the LTNP group (BDL versus LTNP) for CD4+ T cells, the NTHI, JNK MAPK, and granule cell survival pathways are the top three gene sets with FDR < 0.05. In the LTNP group, core enrichment genes in the NTHI pathway were MAP2K3 (MEK3) located along the MAPK p38 cascade and NFKBIA associated with NFKB activation (Figure 6, Additional File 2), indicating the up-regulation of p38 pathway. In JNK MAPK and granule cell survival pathway, MAPK9 (JNK2) and its upstream kinase MAP2K7 (MKK7) were found to be core enrichment genes (Figure 6, Additional File 2), indicating the up-regulation of JNK pathway. Overlapping analysis of core enrichment genes between IGF1, insulin, and NGF pathways (FDR≤0.05) revealed eight common core enrichment genes (GRB2, PIK3R1, PIK3CA, HRAS, MAP2K1, ELK1, JUN, and FOS). These overlapping genes are involved in the ERK signal transduction cascade, another branch of the MAPK signaling pathway (Figure 6). All the aforementioned MAPK associated pathways are also top ranked in the LTNP group in other pairwise comparisons, although they do not reach the highest statistical significance level (Additional File 4).

In the top ranked but less statistically significant gene sets, AKTPATHWAY and WNT signaling pathways are closely associated with cell survival. Comparing VIR versus LTNP for CD4+ T cells, both pathways were enriched in the LTNP group (FDR = 0.23). In the AKTPATHWAY, core enrichment genes included PIK3R1, PIK3CA, and PPP2CA involved in AKT activation, transcription factors associated with cell survival NFKB1, NFKB1A, RELA, FOXO1A, and FOXO3A (Additional File 2). In the WNT pathway, some key genes were detected as core enrichment genes including WNT1, WNT 10A, WNT10B, FZD6, DVL3, TCF7, and CTNNB1 (β-catenin; Additional File 2).

## Discussion

**Figure 7:**
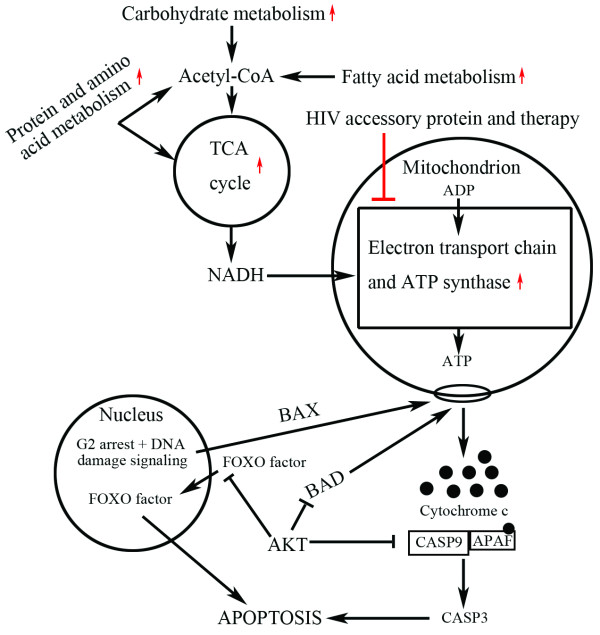
**Schematic overview of the network of enriched pathways related to HIV disease progression**. HIV accessory proteins and side effects of therapy could lead to the impaired activity of electron transport chain complex I, which results in mitochondrial dysfunction. As a compensatory effect, OXPHOS pathway (electron transport chain and ATP synthase) is up-regulated as indicated by the red arrow. Along with the OXPHOS pathway, the TCA cycle supplying NADH to OXPHOS, and a wide range of metabolic pathways (carbohydrate, fatty acid, protein, and amino acid metabolism pathways) furnishing substrates to the TCA cycle are coordinately up-regulated. In addition, mitochondrial dysfunction leads to cell apoptosis mediated by the activation of mitochondrial membrane pore-forming proteins, such as BAX and BAD. Pores generated in the mitochondrial membranes allow the release of the pro-apoptotic proteins cytochrome c, which binds to APAF and hence activates CASP9 leading to the caspase cascade resulting in apoptosis. G2 arrest and DNA damage signaling could also activate BAX leading to mitochondria-mediated apoptosis. On the other hand, AKT could prevent apoptosis by either inhibiting BAD or CASP9 activation or preventing FOXO factors from relocating to the nucleus.

An analysis of the CD4+ and CD8+ T cell transcriptomes from three different HIV disease groups was undertaken to identify gene expression signatures associated with disease progression. All the enriched categories derived from GO enrichment analysis of DE genes corresponded to certain enriched pathways detected by GSEA, confirming the statistical reliability of our analyses. For example, the enriched GO category complement activation corresponded to the enriched pathway HSA04610 complement and coagulation cascades in the comparison of VIR versus BDL/LTNP for CD4+ T cells and the enriched GO category proton-transporting ATPase complex corresponded to the enriched OXPHOS pathway in the VIR versus LTNP comparison for CD8+ cells. Also, GSEA detected more comprehensive pathways correlated with disease progression. Among these enriched pathways, mitochondrial function emerged as a major theme during disease progression as a large portion of the enriched pathways for various physiological processes were all closely associated. These pathways, functionally connected to mitochondria, formed a network directly related to HIV disease progression as discussed below **(**Figure 7**)**.

### Up-regulated metabolic pathways as a transcriptional signature evoked by mitochondria dysfunction in HIV disease progression

Forty-three up-regulated metabolic pathways were detected as transcriptional signatures in the relatively more advanced HIV cases. These signatures were highlighted by the TCA cycle and OXPHOS pathways along with a series of degradation pathways of carbohydrates, fatty acids, and amino acids, articulating with the TCA cycle by furnishing substrates. Interestingly, this comprehensive and unambiguous expression signature in *ex vivo *patient-derived T cells is consistent with two recent CD4+ T cell-line-based proteomic studies which also demonstrated the up-regulation of components of OXPHOS, TCA cycle, amino acid metabolism, and fatty acid metabolism at the protein level in human CD4+ T cell lines after HIV infection[26,27]. The proteomic study, using cell lines, and our transcriptome study, using primary patient cells, are complementary, implying the functional significance of our observations. Further, the detection of the OXPHOS pathway as the most significantly enriched gene set for both CD4+ and CD8+ T cells is also in line with earlier work, which identified OXPHOS pathway up-regulation a distinct transcriptional feature in CD8+ T cells unique to the VIR group when compared against the versus the LTNP group [20]. Moreover, by simultaneously comparing both CD4+ and CD8+ T cells from three HIV disease groups, this study further extends previous findings to provide a panoramic view of all the concordantly regulated metabolic pathways in conjunction with OXPHOS pathway. To our knowledge, this study is the first to show this metabolic transcriptional signature in primary CD4+ and CD8+ T cells, in relation to HIV disease progression. We hypothesize that the up-regulation of these metabolic pathways could be a compensatory event evoked by mitochondrial dysfunction incurred by HIV infection and HAART. This hypothesis is based on the fact that mitochondria are the organelles where the TCA cycle, OXPHOS, and downstream biochemical reactions of degraded products are taking place and mitochondrial dysfunction in HIV disease has been well documented [28]. The NRTIs can inhibit the major mtDNA polymerase [29], induce mtDNA mutations [30], and impair mitochondrial enzymes such as adenylate kinase and the ADP/ATP translocator [31,32]. In addition, the HIV accessory proteins Vpr and Tat, and the HIV protease (Pr) could modulate mitochondrial membrane permeabilization by various pathways involving protein BAX, BAK, BCL-2, and adenine nucleotide translocase (ANT) [33-35].

The hypothesis that metabolic pathways are a compensatory event evoked by mitochondrial dysfunction is further supported by the high similarity between this data and the gene expression profiles from study of compensatory events in primary mitochondrial dysfunction [36]. Using the electron transport chain complex I mutant of *Caenorhabditis elegans *as a model, 29 up-regulated metabolic pathways characterizing the cellular compensatory events accompanying mitochondrial dysfunction were identified [36], of which 15 (> 50%) were shared with our list.

### Pathways involved in mitochondria-mediated cell apoptosis

The significance of mitochondrial dysfunction in HIV disease progression is further strengthened by the detection of coordinately up-regulated genes involved in mitochondria-mediated cell apoptosis in both CD4+ and CD8+ T cells. This is exemplified by the 15 core-enrichment genes in the chemical pathway detected in the CD8+ T cells in the comparison between VIR and BDL groups. For instance, TP53, BAX, and BID could alter the mitochondrial membrane permeability; APAF1 is involved in initiating effector caspase-mediated cell death; CASP3, CASP6, and CASP7 are the effector caspases which cleave substrates leading to modified signaling, and the downstream substrates of these caspases including STAT1, EIF2S1, TLN1 (talin 1), PXN (paxillin), and PRKCB1 (protein kinase C), which eventually lead to cell apoptosis. These genetic level observations were consistent with previous studies at the cellular level showing that the LTNPs had milder mitochondrial impairment and low numbers of cells with reduced mitochondrial membrane potential; this correlates with lower frequency of spontaneous apoptosis and higher frequencies of CD4+ T cells when compared to AIDS patients [37,38].

In addition, the detection of G2 arrest along with the chemical pathway in CD8+ T cells in the VIR group (versus BDL) led to the further speculation that G2 arrest may be functionally linked to mitochondria-mediated cell apoptosis. The HIV protein Vpr induces cell cycle arrest in the G2/M checkpoint in both CD4+ T cells and macrophages [39,40], and there might be a direct correlation between G2 arrest and cell apoptosis [41]. The activation of BAX, the pore-forming mitochondrial protein, has been suggested as the functional linkage between G2 arrest and cell apoptosis [42].

It was unique that in addition to the linkage between G2 arrest and mitochondria-mediated cell apoptosis, the AKT pathway top ranked for the LTNP group (Additional File 4) negatively regulated the chemical pathway by blocking mitochondria-mediated cell apoptosis, which could contribute to cell survival in LTNPs. In the AKT pathway, genes associated with AKT activation including PIK3R1, PIK3CA, and PPP2CA were coordinately up-regulated in the LTNP group (versus VIR) in CD4+ and CD8+ T cells. Activated AKT is known to promote cell survival by phosphorylating BAD and CASP9 to inhibit pore-forming in mitochondria membranes and prevent the subsequent caspase cascade, respectively [43]. Additionally, in the AKT pathway the FOXO factors (FOXO1A, and FOXO3A) detected as core enrichment genes could also contribute to cell survival, as these transcription factors are involved in cell survival [44].

Taken together, a network of pathways closely associated with HIV disease progression was constructed (Figure 7). Centrally located is mitochondrial dysfunction, which interferes with various other pathways ranging from metabolism and energy production to cell cycle dysregulation and mitochondria-meditated cell apoptosis. In addition, the mitochondria-mediated cell apoptosis could be blocked by the AKT pathway enriched in the LTNP group. Consistent with our analysis, two large scale studies of HIV-host interactions by siRNA screening also identified a link between mitochondrial function and HIV replication [45,46]. In addition, Zhou *et al*. have identified the AKT-associated pathway in HIV replication in association with cellular energy metabolism and cell survival, which is well in line with our data.

### MAPK pathway enriched uniquely in the LTNP group

The significant up-regulation of gene sets closely associated with three branches of MAPK pathway (ERK, JNK, and p38) in LTNPs could contribute to cell survival as well as stronger anti-HIV responses. In relation to cell differentiation and activation, JNK and p38 are critical for naïve CD4+ T cell differentiation into the Th1 subset, which antagonizes Th2 subset switch associated with HIV disease progression [3]. Activation of the p38 pathway also results in increased IFN-γ production by both CD4+ and CD8+ T cells, plays an important role in T cell homeostasis by selectively inducing CD8+, but not CD4+ T cell death via modulation of BCL-2 expression [47,48]. ERK is required for Th2 differentiation and the cytotoxic activity of most CD8+ T cells [49]. The ERK pathway could be activated by its upstream signaling pathway, the T-cell receptor pathway, which is also up-regulated in the LTNP group. Supporting this speculation, components of TCR complex CD3epsilon, and ZAP70 (TCR zeta-chain associated protein kinase) as well as genes involved in ERK activation, HRAS (RAS) and MAP2K1 (MEK1), were detected as the core enrichment genes in the T-cell receptor pathway. Further confirmation came from our previous antibody/protein microarray study showing that CD3epsilon expression was significantly higher in LTNP than in the VIR group on CD4+ T cells [20].

Besides direct involvement, the MAPK pathway interacts with a range of pathways critical for cell function and survival, such as p53 and WNT signaling pathways. The top ranked WNT pathway in the LTNP group (Additional File 4) indicated a possible role of this pathway in cell survival; another study has shown that MAPK-p38 pathway regulates WNT-β-catenin signaling [50].

### Critical differences segregating CD4+ and CD8+ T cell transcriptomes during HIV disease

Contrasting with hundreds of differentially expressed genes in CD8+ T cells in the VIR group (versus LTNP), the comparison of BDL versus LTNP revealed only four differentially expressed genes and one enriched gene set, which indicated that the transcriptional profile largely remained unaltered in CD8+ T cells from BDL patients. For the same paired comparison in the CD4+ T cells, although only three differentially expressed genes were detected, 27 enriched gene sets have reached the significance level of FDR < 0.05, which implied the shift of transcriptome profile in CD4+ T cells from BDL patients. Supporting this, an independent study has shown that distinct transcriptional profiles in both CD4+ and CD8+ T cells are established early in HIV infection by comparing between early infection, chronic progressive infection, and non-progression groups [19]. However, the study subjects in this study were grouped by the duration of HIV infection, but not on the plasma HIV load levels (the patients from the early infection group already had detectable viral loads). On the other hand, Hyrcza's data demonstrated that with various viral load levels, even if the infection duration was as short as 1-5 months, the CD8+ T cell transcriptional profile could be shifted. Taken together, these datasets appear to show a close correlation between the beginning of detectable plasma viral load and transcriptome shift uniquely in CD8+ T cells. This was not the case for CD4+ T cell transcriptomes. This study, from the gene and gene set level, further confirmed our recent findings that the CD8+ T cell transcriptome profiles shift only when a viral load is above the certain as yet unknown threshold level [21].

Another transcriptional signature unique to CD4+ T cells is the complement activation in the VIR group (versus BDL/LTNP) detected by both differentially expressed genes analysis and GSEA. This was exemplified by HSA04610 complement and coagulation-cascades pathway; the coordinately up-regulated core enrichment genes include those encoding for the complement proteins and complement receptors (Additional File 3), but not the lytic pathway genes (C6, 7, 8, 9). This is consistent with studies showing that HIV activates the complement cascades, but avoids the lysis via complement regulatory molecules [51,52]. Interactions between HIV envelope protein gp41 and C1Q lead to the complement activation independent of HIV-specific antibodies [53] and the sequentially generated C3 fragments (C3b, iC3b, and C3d) linked to HIV could have high affinity interaction with complement receptors on a wide range of cells such as B cells, macrophages, and follicular dendritic cells [51,54]. In line with these studies, our data strengthens the close association between up-regulated complement activation and HIV disease progression at the genetic level in primary CD4+ T cells, and the impacts of innate immunity on HIV pathogenesis warrants further investigation. Together, these observations imply that different cell subsets differ in their pace towards disease progression and the way they maintain distinct immunologic interaction with HIV during the disease course. Thus, the specific HIV disease stage may bear cell type-specific transcriptomic signatures, which is evident from this study.

## Conclusions

In summary, this study is the first to identify a network of a large panel of pathways functionally connected by mitochondria as a transcriptional signature of HIV disease progression in primary CD4+ and CD8+ T cells. This signature contains 43 metabolic pathways closely articulated with TCA cycle and OXPHOS pathways pointing towards mitochondrial dysfunction. The significance of mitochondrial dysfunction is further strengthened by the detection of coordinately up-regulated genes involved in mitochondria-mediated cell apoptosis in both CD4+ and CD8+ T cells in the VIR group (VIR versus BDL). Mitochondria-mediated cell apoptosis is negatively regulated by the AKT pathway top ranked for the LTNP, which could contribute to cell survival in the LTNPs. Along with the AKT pathway, MAPK and WNT pathways are also closely associated with the LTNPs, which may contribute to cell survival and stronger anti-viral responses via Th1 polarization, IFN-γ regulation, and cytotoxicity activity. Comparisons between CD4+ and CD8+ T cells revealed that the CD8+ T cell transcriptome shifts after the viral load becomes detectable, but this occurs earlier in CD4+ T cell transcriptomes. Another transcriptional signature unique to CD4+ T cells is the complement activation in the VIR group versus BDL/LTNP. Overall, these data offer new comparative insights into HIV disease progression from the aspect of HIV-host interactions at the transcriptomic level, which will facilitate the understanding of the genetic basis of transcriptomic interaction of HIV *in vivo *and how HIV subverts the human gene machinery at the individual cell type level. Further studies on the regulation of these pathways and the corresponding core enrichment genes may provide a detailed understanding of the molecular mechanisms involved, which may also aid the development of therapeutic interventions. Future therapeutic interventions aiming at preserving mitochondrial function could be clinically beneficial. Building up database of the pathway interactions will definitely aid the understanding of the interconnections between various pathways, which will ultimately enable the integration of various molecular mechanisms into a system level.

## Methods

### Patient profiles and collection protocol

Four HIV infected long-term non-progressors (LTNP; n = 4), five HIV+ patients on HAART with below detectable level of plasma viremia (BDL; n = 5), and five viremic HIV+ patients on HAART (VIR; n = 5) along with five healthy HIV seronegative individuals (NEG; n = 5) were studied. No single individual had CCR5-Δ32 homozygous mutation and there is no statistically significant difference in the prevalence of CCR5-Δ32 heterozygous between the study groups. The infection time for L1, L2 and L3 are >20 years, and L4 >14 years. These treatment-naïve LTNPs have maintained high CD4+ T cell counts (> 500 cells/μl) and below detectable plasma viremia (< 50 HIV RNA copies/ml plasma) except one patient (L4) with very low plasma viremia (57 HIV RNA copies/ml plasma) (Table 1). Patients in the VIR group were on HAART and had detectable plasma viremia and CD4+ T cell counts <500 cells/μl, whereas patients in the BDL group showed no detectable viremia while on HAART. These patients received two NRTIs (zidovudine, lamivudine, stavudine, emtricitabine, tenofovir) in association with one or two protease inhibitors (darunavir, ritonavir, indinavir, saquinavir, atazanavir). Eleven patients came from the HIV clinic at Westmead Hospital and three patients plus the five healthy controls came from the Australian Red Cross Blood Service in Sydney. This study was approved by the Sydney West Area Health Services Research Ethics Committee, and all blood samples were collected after individual informed written consent.

### Purification of CD4+ and CD8+ T cells and RNA isolation

A single blood sample (10-20 ml in EDTA) was obtained from each patient. After separation of plasma, PBMC were isolated immediately after obtaining blood samples by Ficoll-gradient centrifugation and purified. This aspect was strictly followed in our experiments because of previously described lower RNA yields and possible changes in gene expression profiles upon storage of blood [55]. CD4+ and CD8+ T cells were then obtained by positive isolation with antibody-conjugated magnetic beads according to the manufacturer's instructions (Dynal Biotech, Oslo, Norway). Flow-cytometric analysis performed on separated CD4+ and CD8+ T cell populations demonstrated that 99.2% ± 0.165 (mean ±SD) and 99.1% ± 0.128 (mean ±SD) of purified CD4+ and CD8+ cells were single positive for the CD4 and CD8 marker, respectively [56]. In positively isolated CD8+ cell population, >97% cells were CD3 positive as shown by flow cytometry in a previous study [18]. Thus, the very low percentage of NK cells in CD8+ cell population would have negligible effect on the results. Total RNA was isolated from purified cells using RNeasy Mini kit (Qiagen Pty Ltd., Clifton Hill, Victoria, Australia) with an integrated step of on-column DNase treatment.

### cRNA preparation, microarray hybridization and scanning

RNA quality was checked by Agilent Bioanalyzer and RNA Integrity Scores are higher than 7 for all the samples. cRNA amplification and labeling with biotin were performed using Illumina TotalPrep RNA amplification kit (Ambion, Inc., Austin, USA) with 250 ng total RNA as input material. cRNA yields were quantified with Agilent Bioanalyzer and 1.5 μg cRNAs were hybridized to the Sentrix Human-6 v2 Expression BeadChips (Illumina, Inc., San Diego, USA). Each chip contains six arrays and each array contains >48,000 gene transcripts, of which, 46,000 derived from human genes in the National Center for Biotechnology Information (NCBI) Reference Sequence (RefSeq) and UniGene databases. All reagents and equipment used for hybridization were purchased from Illumina, Inc. According to the manufacturer's protocol, cRNA was hybridized to arrays for 16 hours at 58°C before being washed and stained with streptavidin-Cy3. Then the beadchips were centrifuged to dry and scanned on the Illumina BeadArray Reader confocal scanner.

### Analysis of differentially expressed genes

The quality of the entire data set was assessed by box plot and density plot of bead intensities, density plot of coefficient of variance, pairwise MAplot, pairwise plot with microarray correlation, cluster dendrogram, and non-metric multidimensional scaling (NMDS) using R/Bioconductor and the lumi package [22]. Based on the quality assessment, all 38 samples were deemed suitable for further analysis. Data normalization was performed using a variance-stabilising transform (VST) and a robust spline normalization (RSN) implemented in the lumi package for R/Bioconductor [22,57]. To reduce false positives, unexpressed genes (based on a detection p value cut-off 0.01) were removed from the dataset. A linear model fit in conjunction with an empirical Bayes statistics were used to identify candidate differentially expressed (DE) genes [23]. Adjustment for multiple testing was performed using the Bonferroni adjustment. For both CD4+ and CD8+ T cells, pairwise comparisons from the 4 study groups (BDL vs NEG, VIR vs NEG, LTNP vs NEG, BDL vs LTNP, VIR vs LTNP, BDL vs VIR) were carried out and candidate DE genes with fold change >2 and B-statistic > 0 were identified for each of the comparisons.

To identify the enriched functional categories from the DE genes, Gene Ontology (GO) Tree from WebGestalt (Web-based Gene SeT AnaLysis Toolkit) was used to identify GO categories with significantly enriched gene numbers [58]. The hypergeometric test was used to calculate the statistic for each category and all genes from human were used as the reference gene set. GO categories with at least 2 genes and p < 0.01 are identified as enriched and colored red in the GOTree. In GOTree, O stands for observed gene number in the category; E for expected gene number in the category; R for ratio of enrichment for the category; and P for p value calculated from the statistical test given for the categories with R > 1 to indicate the significance of enrichment.

### Gene set enrichment analysis

To further understand the biological meanings underlying the transcriptome data from various HIV+ disease groups, a complement approach, gene set enrichment analysis (GSEA) was used [24]. Instead of selecting single DE genes, this method analyzed the entire transcriptome data to identify genes coordinately regulated in predefined gene sets from various biological pathways. For each pairwise comparison (BDL versus LTNP, VIR versus LTNP, BDL versus VIR) for both CD4+ and CD8+ T cells, GSEA was performed using the normalized data of entire 48,000 transcripts (GSEA version 2.0, Broad Institute http://www.broad.mit.edu/gsea). First, a ranked list was obtained by ranking all genes according to the correlation between their expression and the group distinction using the metric signal to noise ratio. Then the association between a given gene set and the group was measured by the non-parametric running sum statistic termed the enrichment score (ES), which was calculated by walking down the ranked list, increasing when encountering a gene in the given gene set and decreasing when encountering a gene not in the gene set. To estimate the statistical significance of the ES, a nominal p value was calculated by permuting the genes 1,000 times. To adjust for multiple hypothesis testing, the maximum ES was normalized to account for the gene set size (NES) and the false discovery rate (FDR) corresponding to each NES was calculated. The gene sets used are from Molecular Signatures Database (MsigDB) [24], catalog C2 functional sets, subcatalog canonical pathways, which include 639 gene sets from pathway databases (version 2.5, updated by April, 2008). These gene sets are canonical representations of a biological process compiled by domain experts such as BioCarta, GenMAPP, and KEGG.

### Real-time quantitative PCR

Purified total cellular RNA was reverse transcribed using oligo d(T) and Superscript III followed by RNase H treatment (Invitrogen Life Technologies). The cDNA was then subject to real-time quantitative PCR with defined primers and SYBR Green (Invitrogen Life Technologies) using Mx3005P™ QPCR System (Stratagene). The relative quantitation method was used to evaluate the expression of selected genes with the housekeeping gene GAPDH as an internal control and the normalizer for all data.

## Competing interests

The authors declare that they have no competing interests.

## Authors' contributions

JQW fully performed the work, analyzed data, and wrote the paper; DED provided the patients, assisted with clinical follow up and details; WBD assisted with LTNP samples, intellectual input with LTNP biology, assistance with writing; YHY assisted with the mathematical and statistical sections; BW assisted with writing and technical aspects of the work and NKS conceived the idea, supervised the work and assisted with writing the manuscript.

## Supplementary Material

Additional file 1 **Differentially expressed genes between HIV+ disease groups**. List of differentially expressed genes between HIV+ disease groups. Click here for file

Additional file 2 **Core enrichment genes in the enriched pathways**. List of core enrichment genes in the enriched pathways. Click here for file

Additional file 3 **Core enrichment genes (highlighted in red) in the complement and coagulation cascade**. Figure of complement and coagulation cascade pathway with highlighted genes. Click here for file

Additional file 4 **Top ranked gene sets enriched in the LTNP group**. List of top ranked gene sets enriched in the LTNP group. Click here for file
