# Supplementary material for: Genome-wide analysis of primary CD4+ and CD8+ T cell transcriptomes shows evidence for a network of enriched pathways associated with HIV disease
Source: Retrovirology. 2011 Mar 16;8:18. doi: 10.1186/1742-4690-8-18 (PMC3068086; doi:10.1186/1742-4690-8-18)
Supplement: Additional file 1 — Differentially expressed genes between HIV+ disease groups. List of differentially expressed genes between HIV+ disease groups. [file 1742-4690-8-18-S1.PDF]

Supplementary file 1: Differentially expressed genes between HIV+ disease groups

Table 1: VIR versus LTNP in CD4+ T cells

Probe	Symbol	GeneID	FC	LogFC	B-statistic	Description
ILMN_18562	FER1L3	26509	2.068483	1.048573	5.23116	fer-1-like 3, myoferlin (C. elegans)
ILMN_14503	IRS2	8660	-2.87394	-1.52303	4.227449	insulin receptor substrate 2
ILMN_20703	CBX4	8535	-2.33526	-1.22358	4.202289	chromobox homolog 4 (Pc class homolog, Drosophila)
ILMN_8881	RAB8A	4218	2.048943	1.03488	4.019728	RAB8A, member RAS oncogene family
ILMN_18066	PRDX1	5052	2.351771	1.233748	4.007767	peroxiredoxin 1
ILMN_27201	RGL1	23179	2.765616	1.467601	3.680638	ral guanine nucleotide dissociation stimulator-like 1
ILMN_29653	PSMB10	5699	2.115736	1.08116	3.400198	proteasome (prosome, macropain) subunit, beta type, 10
ILMN_7981	GPBAR1	151306	2.358469	1.237851	2.621268	G protein-coupled bile acid receptor 1
ILMN_19030	STAT2	6773	2.309794	1.207764	2.569568	signal transducer and activator of transcription 2, 113kDa
ILMN_10935	MOAP1	64112	-2.21843	-1.14954	2.506742	modulator of apoptosis 1
ILMN_18449	PACSIN2	11252	2.132026	1.092225	2.09244	protein kinase C and casein kinase substrate in neurons 2
ILMN_24091	LGALS3E	3959	3.371763	1.753503	2.008359	lectin, galactoside-binding, soluble, 3 binding protein
ILMN_139000	IL8RBP		3.426374	1.776682	1.772684	interleukin 8 receptor, beta pseudogene
ILMN_138176	C1QA	712	7.175437	2.843067	1.51816	complement component 1, q subcomponent, A chain
ILMN_19861	FGL2	10875	3.201163	1.678596	1.512318	fibrinogen-like 2
ILMN_14069	FUCA1	2517	2.085042	1.060077	1.36753	fucosidase, alpha-L- 1, tissue
ILMN_7531	UBE2L6	9246	2.464095	1.301058	1.350643	ubiquitin-conjugating enzyme E2L 6;ubiquitin-conjugating enzyme E2L 6
ILMN_4643	IFI35	3430	3.276368	1.712097	1.348803	interferon-induced protein 35
ILMN_5749	NFE2	4778	2.304499	1.204453	1.22278	nuclear factor (erythroid-derived 2), 45kDa
ILMN_2009	MS4A6A	64231	2.525764	1.33672	1.149959	membrane-spanning 4-domains, subfamily A, member 6A
ILMN_138070	C1QB	713	5.95551	2.574225	1.122734	complement component 1, q subcomponent, B chain
ILMN_5994	OAS2	4939	4.319421	2.110838	1.11482	2'-5'-oligoadenylate synthetase 2, 69/71kDa;2'-5'-oligoadenylate synthetase 2, 69/71kDa
ILMN_12916	PDCD4	27250	-2.32862	-1.21947	1.004964	programmed cell death 4 (neoplastic transformation inhibitor)
ILMN_1807	NR1H3	10062	2.245594	1.167097	1.003168	nuclear receptor subfamily 1, group H, member 3
ILMN_22827	TNFSF10	8743	2.970357	1.570636	0.877446	tumor necrosis factor (ligand) superfamily, member 10
ILMN_14941	SERPING	710	2.770933	1.470372	0.862886	serpin peptidase inhibitor, clade G (C1 inhibitor), member 1, (angioedema, hereditary);serpin peptidase inhibitor, clade G (C1 inhibitor), member 1, (angioedema, hereditary)
ILMN_11175	XAF1	54739	3.938266	1.97756	0.845002	XIAP associated factor-1
ILMN_8937	STAT4	6775	-2.16962	-1.11744	0.661193	signal transducer and activator of transcription 4
ILMN_18780	C1QC	714	4.44271	2.15144	0.650492	complement component 1, q subcomponent, C chain
ILMN_2717	OAS1	4938	3.12547	1.644073	0.495577	2',5'-oligoadenylate synthetase 1, 40/46kDa
ILMN_6174	ISG15	9636	5.121292	2.356508	0.400858	ISG15 ubiquitin-like modifier
ILMN_27504	P2RY10	27334	-2.01969	-1.01413	0.137332	purinergic receptor P2Y, G-protein coupled, 10
ILMN_10713	CECR1	51816	2.026152	1.018742	0.066616	cat eye syndrome chromosome region, candidate 1;cat eye syndrome chromosome region, candidate 1
ILMN_86958	LOC2864	286440	-2.02879	-1.02062	0.06544	hypothetical protein LOC286440
ILMN_26737	ATP6V1D	51382	2.224268	1.15333	0.04049	ATPase, H+ transporting, lysosomal 34kDa, V1 subunit D
ILMN_28175	PRF1	5551	3.200592	1.678339	0.038768	perforin 1 (pore forming protein)

Table 2: BDL versus LTNP in CD4+ T cells

Probe	Symbol	GeneID	FC	LogFC	B-statistic	Description
ILMN_43826	LOC644934		-5.68818	-2.50797	0.481313	hypothetical protein LOC644934
ILMN_46687	hCG_203	644928	-4.69109	-2.22992	0.145381	hCG2033311
ILMN_42423	LOC650298		-3.82598	-1.93583	0.074543	hypothetical protein LOC650298

Table 3: BDL versus VIR in CD4+ T cells

Probe	Symbol	GeneID	FC	LogFC	B-statistic	Description
ILMN_24091	LGALS3E	3959	-3.43119	-1.77871	2.643412	lectin, galactoside-binding, soluble, 3 binding protein
ILMN_19896	BLR1	643	2.001561	1.001125	2.126965	Burkitt lymphoma receptor 1, GTP binding protein (chemokine (C-X-C motif) receptor 5)
ILMN_27201	RGL1	23179	-2.37497	-1.24791	2.070798	ral guanine nucleotide dissociation stimulator-like 1
ILMN_139000	IL8RBP		-3.06248	-1.6147	1.178788	interleukin 8 receptor, beta pseudogene
ILMN_14941	SERPING	710	-2.62342	-1.39145	0.773452	serpin peptidase inhibitor, clade G (C1 inhibitor), member 1, (angioedema, hereditary);serpin peptidase inhibitor, clade G (C1 inhibitor), member 1, (angioedema, hereditary)
ILMN_18780	C1QC	714	-4.0769	-2.02747	0.535159	complement component 1, q subcomponent, C chain
ILMN_46687	hCG_203	644928	-3.54864	-1.82727	0.495803	hCG2033311
ILMN_138070	C1QB	713	-4.78105	-2.25733	0.257128	complement component 1, q subcomponent, B chain
ILMN_16332	MX1	4599	-3.51348	-1.8129	0.000342	myxovirus (influenza virus) resistance 1, interferon-inducible protein p78 (mouse)

Table 4: VIR versus LTNP in CD8+ T cells

Probe	Symbol	GeneID	FC	LogFC	B-statistic	Description
ILMN_43020	LOC642083		2.632611	1.396494	11.7983	hypothetical protein LOC642083
ILMN_3762	KLRD1	3824	2.878025	1.525079	9.648359	killer cell lectin-like receptor subfamily D, member 1
ILMN_16993	LOC442535		2.216706	1.148417	8.506047	hypothetical protein LOC442535
ILMN_27914	LMBR1L	55716	-2.36575	-1.2423	8.104604	limb region 1 homolog (mouse)-like
ILMN_17231	ZNF395	55893	-3.65927	-1.87156	8.05245	zinc finger protein 395
ILMN_37437	BEXL1	56271	-2.63633	-1.39853	7.816819	brain expressed X-linked-like 1
ILMN_5069	PRR5	55615	2.925025	1.548449	6.914539	proline rich 5 (renal)
ILMN_6033	CGRRF1	10668	-2.07355	-1.0521	6.055554	cell growth regulator with ring finger domain 1
ILMN_3307	BATF	10538	2.435663	1.284315	5.769791	basic leucine zipper transcription factor, ATF-like
ILMN_86958	LOC2864	286440	-2.97758	-1.57414	5.747674	hypothetical protein LOC286440
ILMN_14007	CTSC	1075	3.014885	1.592103	5.736425	cathepsin C;cathepsin C
ILMN_29640	STAT1	6772	6.685086	2.740946	5.661323	signal transducer and activator of transcription 1, 91kDa
ILMN_13901	ATP5J2	9551	2.003579	1.002579	5.442544	ATP synthase, H+ transporting, mitochondrial F0 complex, subunit F2
ILMN_23962	GRAMD1	57655	-2.43501	-1.28393	5.312964	GRAM domain containing 1A
ILMN_28413	GBP1	2633	3.23368	1.693177	5.046559	guanylate binding protein 1, interferon-inducible, 67kDa
ILMN_14503	IRS2	8660	-2.98061	-1.57561	4.837575	insulin receptor substrate 2
ILMN_26836	PPP1CA	5499	2.196536	1.13523	4.73724	protein phosphatase 1, catalytic subunit, alpha isoform
ILMN_4753	PSMB2	5690	2.271374	1.183565	4.727512	proteasome (prosome, macropain) subunit, beta type, 2
ILMN_15508	KLRD1	3824	2.308992	1.207263	4.704714	killer cell lectin-like receptor subfamily D, member 1
ILMN_29653	PSMB10	5699	2.259385	1.17593	4.701804	proteasome (prosome, macropain) subunit, beta type, 10
ILMN_2115	LEPREL2	10536	-2.06793	-1.04819	4.642811	leprecan-like 2
ILMN_32029	LOC653103		-2.02722	-1.0195	4.625152	hypothyetical protein LOC653103
ILMN_2565	ACTB	60	2.382926	1.252734	4.591623	actin, beta;actin, beta;actin, beta
ILMN_16255	YIPF4	84272	2.010906	1.007846	4.38509	Yip1 domain family, member 4
ILMN_1146	PYCARD	29108	3.146608	1.653798	4.338386	PYD and CARD domain containing
ILMN_16165	COMMD8	54951	2.082164	1.058084	4.292506	COMM domain containing 8
ILMN_1454	NUDT18	79873	2.071248	1.050501	4.238338	nudix (nucleoside diphosphate linked moiety X)-type motif 18
ILMN_12148	BCL2L1	598	2.133115	1.092962	4.197157	BCL2-like 1;BCL2-like 1
ILMN_4395	NPEPL1	79716	-2.00248	-1.00179	4.172244	aminopeptidase-like 1
ILMN_17789	IL4R	3566	-2.08343	-1.05896	4.147534	interleukin 4 receptor
ILMN_6768	CORO1A	11151	2.213708	1.146465	4.089616	coronin, actin binding protein, 1A
ILMN_137123	WDR74		-2.36722	-1.2432	4.078858	WD repeat domain 74
ILMN_25716	TARP	445347	2.312182	1.209255	4.029385	TCR gamma alternate reading frame protein
ILMN_13489	ARPC5	10092	4.229973	2.080649	4.025077	actin related protein 2/3 complex, subunit 5, 16kDa

ILMN_11054	STAT1	6772	3.560367	1.832026	4.000989	signal transducer and activator of transcription 1, 91kDa;signal transducer and activator of transcription 1, 91kDa
ILMN_9420	BAG3	9531	-2.4162	-1.27274	3.975992	BCL2-associated athanogene 3
ILMN_27043	SUB1	10923	2.279371	1.188636	3.934521	SUB1 homolog (S. cerevisiae)
ILMN_42090	CTA-221(	85379	2.697032	1.431372	3.928604	KIAA1671 protein
ILMN_5404	SESN1	27244	-2.37364	-1.2471	3.863365	sestrin 1
ILMN_17673	FLJ20035	55601	3.065274	1.616016	3.756459	hypothetical protein FLJ20035
ILMN_40116	LOC644063		2.391165	1.257714	3.731178	hypothetical protein LOC644063
ILMN_3776	FBXO6	26270	2.727569	1.447616	3.718484	F-box protein 6
ILMN_21129	CHMP5	51510	2.135013	1.094245	3.514379	chromatin modifying protein 5
ILMN_24427	PRR6	201161	-2.20399	-1.14012	3.468958	proline rich 6
ILMN_6588	ACTA2	59	2.785813	1.478099	3.466841	actin, alpha 2, smooth muscle, aorta
ILMN_13061	RYK	6259	-2.09586	-1.06754	3.415811	RYK receptor-like tyrosine kinase
ILMN_11477	ARL6IP6	151188	2.082116	1.05805	3.402127	ADP-ribosylation-like factor 6 interacting protein 6
ILMN_14812	JARID1B	10765	-2.18107	-1.12504	3.354284	jumonji, AT rich interactive domain 1B
ILMN_27729	FOXJ2	55810	-2.08219	-1.0581	3.350129	forkhead box J2
ILMN_26737	ATP6V1D	51382	2.841399	1.506601	3.193015	ATPase, H+ transporting, lysosomal 34kDa, V1 subunit D
ILMN_8593	CX3CR1	1524	3.031816	1.600182	3.080518	chemokine (C-X3-C motif) receptor 1
ILMN_7535	HERC6	55008	3.189653	1.6734	3.056698	hect domain and RLD 6
ILMN_25394	CCDC53	51019	2.474884	1.307361	3.046174	coiled-coil domain containing 53
ILMN_2354	PFN1	5216	2.265531	1.179849	3.026082	profilin 1
ILMN_10621	CASP1	834	2.616519	1.387649	2.956981	caspase 1, apoptosis-related cysteine peptidase (interleukin 1, beta, convertase)
ILMN_2388	PNPO	55163	3.03704	1.602666	2.927555	pyridoxamine 5'-phosphate oxidase
ILMN_12237	P2RY11	5032	-2.11135	-1.07817	2.843003	purinergic receptor P2Y, G-protein coupled, 11
ILMN_12926	PARP9	83666	3.015748	1.592516	2.825805	poly (ADP-ribose) polymerase family, member 9
ILMN_17355	GADD45/	1647	-2.07751	-1.05485	2.768189	growth arrest and DNA-damage-inducible, alpha
ILMN_30131	OSTF1	26578	2.481112	1.310987	2.72763	osteoclast stimulating factor 1
ILMN_26638	POLR1C	9533	-2.30176	-1.20274	2.721002	polymerase (RNA) I polypeptide C, 30kDa
ILMN_26813	RAC2	5880	2.673716	1.418846	2.70698	ras-related C3 botulinum toxin substrate 2 (rho family, small GTP binding protein Rac2)
ILMN_19572	PSME2	5721	2.309784	1.207758	2.667898	proteasome (prosome, macropain) activator subunit 2 (PA28 beta)
ILMN_5347	NOL11	25926	-2.00377	-1.00271	2.667309	nucleolar protein 11
ILMN_5207	GNG2	54331	2.064496	1.04579	2.663636	guanine nucleotide binding protein (G protein), gamma 2
ILMN_19412	APBB1IP	54518	3.160786	1.660283	2.640836	amyloid beta (A4) precursor protein-binding, family B, member 1 interacting protein
ILMN_19806	C4orf20	55325	2.190115	1.131007	2.613663	chromosome 4 open reading frame 20
ILMN_16589	ACADM	34	2.124768	1.087306	2.576618	acyl-Coenzyme A dehydrogenase, C-4 to C-12 straight chain
ILMN_19977	DKFZp68	374383	-2.00271	-1.00196	2.536222	hypothetical protein DKFZp686O24166
ILMN_138022	TSPYL2		-2.46708	-1.3028	2.527756	TSPY-like 2
ILMN_27754	EPSTI1	94240	6.020071	2.58978	2.519521	epithelial stromal interaction 1 (breast)
ILMN_2973	RNF167	26001	2.231123	1.15777	2.45923	ring finger protein 167
ILMN_30038	RAB7A	7879	2.550865	1.350986	2.454973	RAB7A, member RAS oncogene family
ILMN_1682	M6PR	4074	2.3142	1.210513	2.427404	mannose-6-phosphate receptor (cation dependent)
ILMN_138894	SCML1	6322	-2.81198	-1.49159	2.358354	sex comb on midleg-like 1 (Drosophila)
ILMN_24933	MRPL48	51642	2.008283	1.005963	2.347245	mitochondrial ribosomal protein L48
ILMN_96342	TRIM69	140691	2.463928	1.30096	2.330309	tripartite motif-containing 69
ILMN_1415	GPR114	221188	2.612226	1.38528	2.314722	G protein-coupled receptor 114
ILMN_5994	OAS2	4939	5.020285	2.327769	2.286667	2'-5'-oligoadenylate synthetase 2, 69/71kDa;2'-5'-oligoadenylate synthetase 2, 69/71kDa
ILMN_26206	GTF2H5	404672	2.419603	1.27477	2.251579	general transcription factor IIH, polypeptide 5
ILMN_35118	LOC4007:	400759	2.009911	1.007132	2.225074	similar to Interferon-induced guanylate-binding protein 1 (GTP-binding protein 1) (Guanine nucleotide-binding protein 1) (HuGBP-1)
ILMN_9634	YWHAB	7529	2.440029	1.286899	2.171997	tyrosine 3-monooxygenase/tryptophan 5-monooxygenase activation protein, beta polypeptide
ILMN_9157	UNC119	9094	-2.10688	-1.07511	2.155703	unc-119 homolog (C. elegans)
ILMN_15259	PSMA5	5686	2.672809	1.418357	2.075958	proteasome (prosome, macropain) subunit, alpha type, 5
ILMN_22965	LYST	1130	2.165331	1.114587	2.049219	lysosomal trafficking regulator
ILMN_12139	PSMB8	5696	2.3127	1.209578	2.049062	proteasome (prosome, macropain) subunit, beta type, 8 (large multifunctional peptidase 7)
ILMN_13248	TPST2	8459	2.389734	1.25685	2.021624	tyrosylprotein sulfotransferase 2
ILMN_7457	CDK2AP2	10263	2.00431	1.003106	1.998445	CDK2-associated protein 2
ILMN_18834	ASCC3L1	23020	-2.18732	-1.12917	1.993594	activating signal cointegrator 1 complex subunit 3-like 1
ILMN_2749	GBP4	115361	2.592615	1.374408	1.962922	guanylate binding protein 4
ILMN_24804	KIAA032:	23351	-2.18926	-1.13044	1.915335	KIAA0323
ILMN_22938	TMBIM4	51643	2.183703	1.126777	1.824918	transmembrane BAX inhibitor motif containing 4
ILMN_18673	LY6E	4061	2.863558	1.517809	1.822137	lymphocyte antigen 6 complex, locus E
ILMN_22580	REM2	161253	-2.11079	-1.07779	1.818409	RAS (RAD and GEM)-like GTP binding 2
ILMN_2202	ME2	4200	2.032782	1.023456	1.765436	malic enzyme 2, NAD(+)-dependent, mitochondrial
ILMN_7338	MRPL51	51258	2.69807	1.431928	1.746922	mitochondrial ribosomal protein L51
ILMN_21943	SLAMF6	114836	2.349544	1.232381	1.737355	SLAM family member 6
ILMN_19519	CD53	963	2.344005	1.228976	1.732349	CD53 molecule
ILMN_16128	FASN	2194	-2.64929	-1.40561	1.702752	fatty acid synthase
ILMN_12629	FBXL11	22992	-2.21288	-1.14592	1.683348	F-box and leucine-rich repeat protein 11
ILMN_4643	IFI35	3430	3.387238	1.760109	1.661528	interferon-induced protein 35
ILMN_8923	ATP6V0E	8992	2.04237	1.030244	1.658751	ATPase, H+ transporting, lysosomal 9kDa, V0 subunit e1
ILMN_8513	VKORC1	79001	2.020451	1.014678	1.632027	vitamin K epoxide reductase complex, subunit 1;vitamin K epoxide reductase complex, subunit 1
ILMN_16347	SH3BGRI	6451	2.002348	1.001692	1.618374	SH3 domain binding glutamic acid-rich protein like
ILMN_22873	ALG9	79796	-2.40232	-1.26443	1.552164	asparagine-linked glycosylation 9 homolog (S. cerevisiae, alpha- 1,2-mannosyltransferase)
ILMN_18826	TMEM12(	55863	2.250431	1.170201	1.538283	transmembrane protein 126B
ILMN_23486	SNF1LK	150094	-2.98148	-1.57603	1.517037	SNF1-like kinase
ILMN_1275	ZMYM6	9204	3.927343	1.973554	1.500123	zinc finger, MYM-type 6
ILMN_10698	GALM	130589	2.168751	1.116865	1.475404	galactose mutarotase (aldose 1-epimerase)
ILMN_13962	CAT	847	2.158241	1.109856	1.462729	catalase
ILMN_43942	LOC6538:	653888	2.549373	1.350142	1.445212	similar to Actin-related protein 2/3 complex subunit 1B (ARP2/3 complex 41 kDa subunit) (p41-ARC)
ILMN_121109			-2.42612	-1.27865	1.411488	
ILMN_3023	HN1	51155	2.199467	1.137154	1.398873	hematological and neurological expressed 1
ILMN_6497	ITGA5	3678	-3.40758	-1.76875	1.392089	integrin, alpha 5 (fibronectin receptor, alpha polypeptide)
ILMN_27077	NECAP1	25977	-2.35865	-1.23796	1.389148	NECAP endocytosis associated 1
ILMN_6622	PTGER2	5732	2.768219	1.468958	1.373735	prostaglandin E receptor 2 (subtype EP2), 53kDa
ILMN_3703	FBXO33	254170	-2.14781	-1.10287	1.342942	F-box protein 33
ILMN_15857	ABI3	51225	2.382068	1.252215	1.338873	ABI gene family, member 3
ILMN_25729	MRCL3	10627	2.151637	1.105435	1.318874	myosin regulatory light chain MRCL3
ILMN_18051	ASCL2	430	2.492094	1.317359	1.262396	achaete-scute complex homolog 2 (Drosophila)
ILMN_887	ARL6IP5	10550	2.086168	1.060855	1.258347	ADP-ribosylation-like factor 6 interacting protein 5
ILMN_19750	YPEL2	388403	-2.60106	-1.3791	1.253813	yippee-like 2 (Drosophila)
ILMN_137691	RGS19	10287	2.335561	1.223769	1.246707	regulator of G-protein signaling 19
ILMN_6272	DUSP23	54935	2.266135	1.180234	1.203226	dual specificity phosphatase 23
ILMN_3760	HSF2	3298	-2.1692	-1.11716	1.150273	heat shock transcription factor 2

ILMN_8084	TMCO1	54499	2.157677	1.109479	1.137738	transmembrane and coiled-coil domains 1
ILMN_2717	OAS1	4938	3.360602	1.74872	1.137341	2',5'-oligoadenylate synthetase 1, 40/46kDa
ILMN_2393	FKSG30	440915	3.190224	1.673658	1.096679	kappa-actin
ILMN_25774	KLRB1	3820	-3.70662	-1.89011	1.076823	killer cell lectin-like receptor subfamily B, member 1
ILMN_12613	KLRG1	10219	3.388749	1.760753	1.052591	killer cell lectin-like receptor subfamily G, member 1
ILMN_11175	XAF1	54739	4.056099	2.020093	1.048699	XIAP associated factor-1
ILMN_506	BTBD11	121551	-2.43644	-1.28477	1.040698	BTB (POZ) domain containing 11;BTB (POZ) domain containing 11
ILMN_19030	STAT2	6773	2.088294	1.062325	1.002877	signal transducer and activator of transcription 2, 113kDa
ILMN_75486			2.494722	1.318879	0.999828	
ILMN_85308			-2.24623	-1.16751	0.940687	
ILMN_28175	PRF1	5551	3.559762	1.831781	0.916104	perforin 1 (pore forming protein)
ILMN_6174	ISG15	9636	5.568522	2.477295	0.897567	ISG15 ubiquitin-like modifier
ILMN_21424	C6orf125	84300	2.071111	1.050405	0.87017	chromosome 6 open reading frame 125
ILMN_21536	BSG	682	2.284381	1.191803	0.834209	basigin (Ok blood group)
ILMN_23872	USP36	57602	-3.12289	-1.64288	0.818454	ubiquitin specific peptidase 36
ILMN_14549	C13orf15	28984	-4.49673	-2.16888	0.771176	chromosome 13 open reading frame 15
ILMN_36935	MAST3	23031	2.149912	1.104278	0.767063	microtubule associated serine/threonine kinase 3
ILMN_9886	PTPN6		2.056576	1.040244	0.762786	protein tyrosine phosphatase, non-receptor type 6
ILMN_112467			-2.53843	-1.34394	0.748175	
ILMN_3399	C12orf41	54934	-2.20357	-1.13985	0.742712	chromosome 12 open reading frame 41
ILMN_27133	SAMD9L	219285	3.380122	1.757075	0.73407	sterile alpha motif domain containing 9-like
ILMN_29174	RWDD1	51389	-2.09664	-1.06808	0.700654	RWD domain containing 1
ILMN_19845	STK39	27347	2.175245	1.121178	0.69472	serine threonine kinase 39 (STE20/SPS1 homolog, yeast)
ILMN_995	EXOSC3	51010	2.000818	1.00059	0.686399	exosome component 3
ILMN_3024	RNF20	56254	2.079975	1.056566	0.6834	ring finger protein 20
ILMN_15855	ARFIP1	27236	2.066933	1.047492	0.66766	ADP-ribosylation factor interacting protein 1 (arfaptin 1)
ILMN_7531	UBE2L6	9246	2.341155	1.227221	0.659313	ubiquitin-conjugating enzyme E2L 6;ubiquitin-conjugating enzyme E2L 6
ILMN_9752	IFI44L	10964	9.157134	3.194896	0.628646	interferon-induced protein 44-like
ILMN_23081	PLEKHO	51177	-2.62554	-1.39262	0.60974	pleckstrin homology domain containing, family O member 1
ILMN_26580	PCMT1	5110	3.713247	1.892681	0.573802	protein-L-isoaspartate (D-aspartate) O-methyltransferase
ILMN_9901	PGRMC2	10424	-2.08725	-1.0616	0.556677	progesterone receptor membrane component 2
ILMN_7749	GRPEL1	80273	-2.02547	-1.01826	0.545702	GrpE-like 1, mitochondrial (E. coli)
ILMN_15689	OAS3	4940	2.816218	1.493759	0.503593	2'-5'-oligoadenylate synthetase 3, 100kDa
ILMN_8205	TMEM41	440026	-2.1245	-1.08712	0.495852	transmembrane protein 41B
ILMN_18040	GALK1	2584	2.068378	1.0485	0.484609	galactokinase 1
ILMN_9871	PARP10	84875	2.377608	1.249511	0.476797	poly (ADP-ribose) polymerase family, member 10;poly (ADP-ribose) polymerase family, member 10
ILMN_10231	AIM2	9447	2.330371	1.22056	0.47622	absent in melanoma 2
ILMN_9027	ZMAT3	64393	2.159401	1.110631	0.451759	zinc finger, matrin type 3
ILMN_20286	NDUFB5	4711	2.156949	1.108992	0.449978	NADH dehydrogenase (ubiquinone) 1 beta subcomplex, 5, 16kDa
ILMN_25398	FLJ14213	79899	2.322431	1.215635	0.433792	hypothetical protein FLJ14213
ILMN_22884	ZNF317	57693	-2.09935	-1.06994	0.419005	zinc finger protein 317
ILMN_2834	DYNC1L	1783	-2.03986	-1.02847	0.39519	dynein, cytoplasmic 1, light intermediate chain 2
ILMN_10283	CDR2	1039	-2.26916	-1.18216	0.390047	cerebellar degeneration-related protein 2, 62kDa
ILMN_1431	RALBP1	10928	2.079594	1.056302	0.297277	ralA binding protein 1
ILMN_14090	IQGAP1	8826	2.562804	1.357723	0.279485	IQ motif containing GTPase activating protein 1
ILMN_7185	SLAMF7	57823	2.029755	1.021306	0.259062	SLAM family member 7
ILMN_33294	ZNF815	401303	-2.014	-1.01007	0.235615	zinc finger protein 815
ILMN_23615	HIP1R	9026	-2.40353	-1.26516	0.2019	huntingtin interacting protein 1 related;huntingtin interacting protein 1 related
ILMN_23042	IFRD1	3475	-2.14576	-1.10149	0.160659	interferon-related developmental regulator 1
ILMN_11095	CSNK1D	1453	-2.96903	-1.56999	0.154278	casein kinase 1, delta;casein kinase 1, delta
ILMN_9410	SNIP1	79753	-3.32293	-1.73246	0.153871	Smad nuclear interacting protein 1
ILMN_15905	IKZF5	64376	-2.11765	-1.08247	0.129999	IKAROS family zinc finger 5 (Pegasus)
ILMN_23945	KIAA083	22863	-2.11044	-1.07755	0.129231	KIAA0831
ILMN_40435	LOC6430	643031	-2.10954	-1.07693	0.116164	similar to NADH dehydrogenase subunit 5
ILMN_16811	PER1	5187	-2.0295	-1.02112	0.056718	period homolog 1 (Drosophila)
ILMN_26277	DENND2	79961	3.412441	1.770804	0.046393	DENN/MADD domain containing 2D

Table 5: BDL versus LTNP in CD8+ T cells

Probe	Symbol	GeneID	FC	LogFC	B-statistic	Description
ILMN_40116	LOC644063		2.459872	1.298583	4.03941	hypothetical protein LOC644063
ILMN_38337	LOC653994		2.170378	1.117946	3.304621	hypothetical protein LOC653994
ILMN_3762	KLRD1	3824	2.050351	1.035871	3.10808	killer cell lectin-like receptor subfamily D, member 1
ILMN_138022	TSPYL2		-2.07619	-1.05394	0.104315	TSPY-like 2

Table 6: BDL versus VIR in CD8+ T cells

Probe	Symbol	GeneID	FC	LogFC	B-statistic	Description
ILMN_14503	IRS2	8660	2.732926	1.450446	4.283349	insulin receptor substrate 2
ILMN_17231	ZNF395	55893	2.291685	1.196408	2.003257	zinc finger protein 395
ILMN_40435	LOC6430	643031	2.277432	1.187408	1.875407	similar to NADH dehydrogenase subunit 5
ILMN_12926	PARP9	83666	-2.60949	-1.38377	1.740943	poly (ADP-ribose) polymerase family, member 9
ILMN_16332	MX1	4599	-4.28586	-2.09959	1.632645	myxovirus (influenza virus) resistance 1, interferon-inducible protein p78 (mouse)
ILMN_17673	FLJ20035	55601	-2.44161	-1.28783	1.506975	hypothetical protein FLJ20035
ILMN_28413	GBP1	2633	-2.3565	-1.23665	1.485222	guanylate binding protein 1, interferon-inducible, 67kDa
ILMN_137396	CD79A	973	2.224849	1.153707	1.095849	CD79a molecule, immunoglobulin-associated alpha;CD79a molecule, immunoglobulin-associated alpha
ILMN_14007	CTSC	1075	-2.07318	-1.05184	0.852278	cathepsin C;cathepsin C
ILMN_76085			-2.12206	-1.08546	0.827091	
ILMN_6588	ACTA2	59	-2.18419	-1.1271	0.798084	actin, alpha 2, smooth muscle, aorta
ILMN_2717	OAS1	4938	-2.97454	-1.57267	0.638917	2',5'-oligoadenylate synthetase 1, 40/46kDa
ILMN_7535	HERC6	55008	-2.41065	-1.26942	0.439762	hect domain and RLD 6
ILMN_15857	ABI3	51225	-2.12204	-1.08545	0.436107	ABI gene family, member 3
ILMN_5994	OAS2	4939	-3.6131	-1.85324	0.346756	2'-5'-oligoadenylate synthetase 2, 69/71kDa;2'-5'-oligoadenylate synthetase 2, 69/71kDa
ILMN_2504	AOAH	313	-2.36467	-1.24164	0.094761	acyloxyacyl hydrolase (neutrophil)
ILMN_35931	FAM139B	653691	2.066442	1.047149	0.036343	family with sequence similarity 139, member B

FC: fold change
