# Supplementary material for: Genome-wide analysis of primary CD4+ and CD8+ T cell transcriptomes shows evidence for a network of enriched pathways associated with HIV disease
Source: Retrovirology. 2011 Mar 16;8:18. doi: 10.1186/1742-4690-8-18 (PMC3068086; doi:10.1186/1742-4690-8-18)
Supplement: Additional file 2 — Core enrichment genes in the enriched pathways. List of core enrichment genes in the enriched pathways. [file 1742-4690-8-18-S2.PDF]

Supplementary file 2: List of core enrichment genes

Table I: Core enrichment genes in chemical pathway in CD8+ T cells from the VIR group (VIR versus BDL)

GENE SYMBOL	GENE_TITLE	RANK IN GENE LIST	RANK METRIC SCORE[b]	RUNNING ES[c]	CORE ENRICHMENT[d]
STAT1	signal transducer and activator of transcription 1, 91kDa	53	0.249643385	0.1654707	Yes
BCL2L1	BCL2-like 1	444	0.127311036	0.24127091	Yes
CASP7	caspase 7, apoptosis-related cysteine peptidase	582	0.113584325	0.3138878	Yes
TLN1	talin 1	748	0.103273571	0.37895638	Yes
EIF2S1	eukaryotic translation initiation factor 2, subunit 1 alpha, 35kDa	890	0.095142022	0.439162	Yes
BCL2	B-cell CLL/lymphoma 2	914	0.093827918	0.5012809	Yes
APAF1	apoptotic peptidase activating factor	1250	0.078865506	0.54602766	Yes
BID	BH3 interacting domain death agonist	1603	0.067316748	0.5826595	Yes
BAX	BCL2-associated X protein	2283	0.051061049	0.6007006	Yes
CASP6	caspase 6, apoptosis-related cysteine peptidase	2679	0.043630809	0.62049675	Yes
PXN	paxillin	2749	0.042432118	0.647203	Yes
CASP3	caspase 3, apoptosis-related cysteine peptidase	2866	0.040523142	0.67152274	Yes
PRKCB1	protein kinase C, beta 1	3115	0.036747769	0.6901989	Yes
TP53	tumor protein p53 (Li-Fraumeni syndrome)	3242	0.034944672	0.7105565	Yes
AKT1	v-akt murine thymoma viral oncogene homolog 1	3303	0.034272321	0.7320261	Yes
BAD	BCL2-antagonist of cell death	9700	0.009734289	0.58724636	No
CYCS	cytochrome c, somatic	19152	0.001647748	0.36480793	No
CASP9	caspase 9, apoptosis-related cysteine peptidase	32773	-0.009190801	0.048800267	No
ATM	ataxia telangiectasia mutated (includes complementation groups A, C and D)	37017	-0.017306691	-0.039998613	No
PTK2	PTK2 protein tyrosine kinase 2	39416	-0.038237713	-0.07118003	No
PRKCA	protein kinase C, alpha	42224	-0.208649144	0.001773955	No

Table II: Core enrichment genes in apoptosis pathway in CD4+ T cells from the VIR group (VIR versus LTNP)

GENE SYMBOL	GENE_TITLE	RANK IN GENE LIST	RANK METRIC SCORE	RUNNING ES	CORE ENRICHMENT
PRF1	perforin 1 (pore forming protein)	13	0.460867912	0.10043812	Yes
TNFSF10	tumor necrosis factor (ligand) superfamily, member 10	25	0.383988917	0.18411781	Yes
GZMB	granzyme B (granzyme 2, cytotoxic T-lymphocyte-associated serine esterase 1)	52	0.310286254	0.25133088	Yes
IRF7	interferon regulatory factor 7	65	0.29074195	0.31460306	Yes
CASP1	caspase 1, apoptosis-related cysteine peptidase (interleukin 1, beta, convertase)	209	0.20282419	0.35555464	Yes
TNFRSF1A	tumor necrosis factor receptor superfamily, member 1A	331	0.17348285	0.39061308	Yes
BCL2L1	BCL2-like 1	687	0.131710097	0.4109994	Yes
NFKBIE	nuclear factor of kappa light polypeptide gene enhancer in B-cells inhibitor, epsilon	810	0.12193127	0.43476495	Yes
RIPK1	receptor (TNFRSF)-interacting serine-threonine kinase 1	1234	0.094939239	0.4455031	Yes
CASP7	caspase 7, apoptosis-related cysteine peptidase	1239	0.094826832	0.46613756	Yes
TNFRSF1B	tumor necrosis factor receptor superfamily, member 1B	1518	0.083671212	0.47784576	Yes
CASP6	caspase 6, apoptosis-related cysteine peptidase	1739	0.076533347	0.48936692	Yes
DFFA	DNA fragmentation factor, 45kDa, alpha polypeptide	1973	0.06954705	0.49905303	Yes
IKBKB	inhibitor of kappa light polypeptide gene enhancer in B-cells, kinase beta	2070	0.066938594	0.5114128	Yes
MAP3K1	mitogen-activated protein kinase kinase kinase 1	2124	0.065570541	0.5244916	Yes
DFFB	DNA fragmentation factor, 40kDa, beta polypeptide (caspase-activated DNase)	2137	0.065164194	0.53845245	Yes
MYC	v-myc myelocytomatosis viral oncogene homolog (avian)	2163	0.06464649	0.55199224	Yes
IRF4	interferon regulatory factor 4	2480	0.055935305	0.5567376	Yes
BCL2	B-cell CLL/lymphoma 2	3124	0.042706084	0.5508485	Yes
TRAF2	TNF receptor-associated factor 2	3142	0.04243407	0.55972207	Yes
BID	BH3 interacting domain death agonist	3164	0.042057045	0.5684185	Yes
IRF1	interferon regulatory factor 1	3306	0.03965817	0.57374924	Yes
MAP2K4	mitogen-activated protein kinase kinase 4	3417	0.037493601	0.5793408	Yes
TRADD	TNFRSF1A-associated via death domain	3418	0.037492443	0.5875367	Yes
IRF2	interferon regulatory factor 2	3564	0.035024107	0.59175974	Yes
IKBKG	inhibitor of kappa light polypeptide gene enhancer in B-cells, kinase gamma	4090	0.027931774	0.5854349	No
BAX	BCL2-associated X protein	4215	0.026773736	0.5883516	No
BIRC4	baculoviral IAP repeat-containing 4	4803	0.022195242	0.57930475	No
CASP9	caspase 9, apoptosis-related cysteine peptidase	5019	0.021175863	0.5788431	No
CASP4	caspase 4, apoptosis-related cysteine peptidase	5292	0.019903162	0.5767537	No
IRF3	interferon regulatory factor 3	5678	0.018331503	0.571645	No
FAS	Fas (TNF receptor superfamily, member 6)	6331	0.016303813	0.55977124	No
TP53	tumor protein p53 (Li-Fraumeni syndrome)	6922	0.014929856	0.5490652	No
CASP10	caspase 10, apoptosis-related cysteine peptidase	6926	0.01491113	0.5522537	No
HELLS	helicase, lymphoid-specific	7145	0.014414215	0.55024296	No
CASP3	caspase 3, apoptosis-related cysteine peptidase	9959	0.010074225	0.48584005	No
HRK	harakiri, BCL2 interacting protein (contains only BH3 domain)	13987	0.005965762	0.39179447	No
BIRC5	baculoviral IAP repeat-containing 5 (survivin)	14811	0.005269074	0.3734596	No
FASLG	Fas ligand (TNF superfamily, member 6)	18683	0.002288961	0.282304	No
IRF6	interferon regulatory factor 6	19156	0.001950088	0.27155444	No
TP73	tumor protein p73	21390	3.22E-04	0.21875273	No
TRAF3	TNF receptor-associated factor 3	23207	-9.91E-04	0.17597076	No
IRF5	interferon regulatory factor 5	26050	-0.003177433	0.1093736	No
LTA	lymphotoxin alpha (TNF superfamily, member 1)	26598	-0.003606332	0.097210295	No
CASP2	caspase 2, apoptosis-related cysteine peptidase (neural precursor cell expressed, developmentally down-regulated 2)	27940	-0.004688888	0.066483624	No
TNFRSF21	tumor necrosis factor receptor superfamily, member 21	28261	-0.004944195	0.05998759	No
MAPK10	mitogen-activated protein kinase 10	29095	-0.00564577	0.041498315	No
BAD	BCL2-antagonist of cell death	29632	-0.006159219	0.030153526	No
CHUK	conserved helix-loop-helix ubiquitous kinase	31397	-0.007945098	-0.009876967	No
MDM2	Mdm2, transformed 3T3 cell double minute 2, p53 binding protein (mouse)	32298	-0.009011963	-0.029216792	No
CYCS	cytochrome c, somatic	32434	-0.009150354	-0.030412998	No
TNFRSF10B	tumor necrosis factor receptor superfamily, member 10b	36364	-0.01668543	-0.11979487	No
FADD	Fas (TNFRSF6)-associated via death domain	36495	-0.01713956	-0.11912624	No
NFKBIB	nuclear factor of kappa light polypeptide gene enhancer in B-cells inhibitor, beta	37831	-0.024146097	-0.14545749	No
TRAF1	TNF receptor-associated factor 1	38403	-0.029593853	-0.15250818	No
NFKB1	nuclear factor of kappa light polypeptide gene enhancer in B-cells 1 (p105)	39571	-0.048017658	-0.16964327	No
NFKBIA	nuclear factor of kappa light polypeptide gene enhancer in B-cells inhibitor, alpha	39626	-0.049282972	-0.16014858	No
BIRC2	baculoviral IAP repeat-containing 2	40241	-0.062981032	-0.16091894	No
BIRC3	baculoviral IAP repeat-containing 3	40357	-0.066497765	-0.14910543	No
RELA	v-rel reticuloendotheliosis viral oncogene homolog A, nuclear factor of kappa light polypeptide gene enhancer in B-cells 3, p65 (avian)	40830	-0.081437863	-0.1424789	No
BCL2L11	BCL2-like 11 (apoptosis facilitator)	41015	-0.088877454	-0.12740694	No
CASP8	caspase 8, apoptosis-related cysteine peptidase	41242	-0.101338267	-0.110605486	No
BNIP3L	BCL2/adenovirus E1B 19kDa interacting protein 3-like	41456	-0.114566945	-0.09060443	No
TNF	tumor necrosis factor (TNF superfamily, member 2)	41677	-0.130077586	-0.06737848	No
TNFRSF25	tumor necrosis factor receptor superfamily, member 25	41829	-0.144271314	-0.039416034	No
JUN	jun oncogene	42219	-0.231110632	0.001894263	No

Table III: Core enrichment genes in NTHI pathway in CD4+ T cells from the LTNP group (BDL versus LTNP)

GENE SYMBOL	GENE_TITLE	RANK IN GENE LIST	RANK METRIC SCORE	RUNNING ES	CORE ENRICHMENT
IKBKB	inhibitor of kappa light polypeptide gene enhancer in B-cells, kinase beta	1581	0.050756909	-0.003242652	No
NR3C1	nuclear receptor subfamily 3, group C, member 1 (glucocorticoid receptor)	4554	0.02081091	-0.05953627	No
MAPK11	mitogen-activated protein kinase 11	9187	0.011287683	-0.16150168	No
MYD88	myeloid differentiation primary response gene (88)	19545	0.002014488	-0.40511993	No
MAPK14	mitogen-activated protein kinase 14	21823	4.27E-04	-0.4586907	No
TLR2	toll-like receptor 2	23075	-5.08E-04	-0.48793906	No
MAP3K14	mitogen-activated protein kinase kinase kinase 14	31812	-0.00771788	-0.6893782	No
MAP2K6	mitogen-activated protein kinase kinase 6	32243	-0.008184629	-0.69404185	No

TGFBR1	transforming growth factor, beta receptor I (activin A receptor type II-like kinase, 53kDa)	35458	-0.012754697	-0.7614802	No
NFKB1	nuclear factor of kappa light polypeptide gene enhancer in B-cells 1 (p105)	36629	-0.015346538	-0.778828	No
CHUK	conserved helix-loop-helix ubiquitous kinase	37561	-0.018262053	-0.788561	No
MAP3K7	mitogen-activated protein kinase kinase kinase 7	38109	-0.020746259	-0.78753966	No
MAP2K3	mitogen-activated protein kinase kinase 3	39481	-0.031773143	-0.79858875	Yes
NFKBIA	nuclear factor of kappa light polypeptide gene enhancer in B-cells inhibitor, alpha	40998	-0.058815632	-0.7948715	Yes
EP300	E1A binding protein p300	41606	-0.079600103	-0.7556685	Yes
TNF	tumor necrosis factor (TNF superfamily, member 2)	41804	-0.090466224	-0.69945633	Yes
CREBBP	CREB binding protein (Rubinstein-Taybi syndrome)	41866	-0.095700502	-0.63650537	Yes
RELA	v-rel reticuloendotheliosis viral oncogene homolog A, nuclear factor of kappa light polypeptide gene enhancer in B-cells 3, p65 (avian)	41871	-0.096471459	-0.57168746	Yes
TGFBR2	transforming growth factor, beta receptor II (70/80kDa)	42070	-0.118444145	-0.49667352	Yes
DUSP1	dual specificity phosphatase 1	42239	-0.174744323	-0.3830674	Yes
IL1B	interleukin 1, beta	42268	-0.223157927	-0.23357393	Yes
IL8	interleukin 8	42288	-0.348186731	2.60E-04	Yes

Table IV: Core enrichment genes in ST_JNK_MAPK pathway in CD4+ T cells from the LTNP group (BDL versus LTNP)

GENE SYMBOL	GENE_TITLE	RANK IN GENE LIST	RANK METRIC SCORE	RUNNING ES	CORE ENRICHMENT
ZAK	-	1323	0.057003167	-7.62E-05	No
MAP3K12	mitogen-activated protein kinase kinase kinase 12	1826	0.045219075	0.012818848	No
MAP3K11	mitogen-activated protein kinase kinase kinase 11	1932	0.043082654	0.033937667	No
MAP2K5	mitogen-activated protein kinase kinase 5	1994	0.042110752	0.05556519	No
MAP3K1	mitogen-activated protein kinase kinase kinase 1	2803	0.031036031	0.05344898	No
MAP3K7IP1	mitogen-activated protein kinase kinase kinase 7 interacting protein 1	3402	0.026384087	0.053753376	No
DUSP8	dual specificity phosphatase 8	4596	0.020605091	0.03681216	No
SHC1	SHC (Src homology 2 domain containing) transforming protein 1	4684	0.020332368	0.04589285	No
MAPK8	mitogen-activated protein kinase 8	5502	0.01787206	0.036351606	No
MAP3K4	mitogen-activated protein kinase kinase kinase 4	8308	0.012500482	-0.023174653	No
MYEF2	myelin expression factor 2	11104	0.009103092	-0.08432559	No
MAP2K4	mitogen-activated protein kinase kinase 4	11675	0.008515513	-0.09314818	No
MAP3K13	mitogen-activated protein kinase kinase kinase 13	15013	0.00546591	-0.16911717	No
MAP3K5	mitogen-activated protein kinase kinase kinase 5	18580	0.002721158	-0.25200874	No
AKT1	v-akt murine thymoma viral oncogene homolog 1	22446	-3.94E-05	-0.3434448	No
DLD	dihydrolipoamide dehydrogenase	30504	-0.006429035	-0.53057563	No
IL1R1	interleukin 1 receptor, type I	30505	-0.006429067	-0.5270534	No
GAB1	GRB2-associated binding protein 1	31625	-0.007506792	-0.54941964	No
MAPK10	mitogen-activated protein kinase 10	31743	-0.007643675	-0.5480005	No
PAPPA	pregnancy-associated plasma protein A, pappalysin 1	32029	-0.007940724	-0.55039406	No
MAP3K10	mitogen-activated protein kinase kinase kinase 10	32283	-0.008243318	-0.55186456	No
GCK	glucokinase (hexokinase 4, maturity onset diabetes of the young 2)	34152	-0.010562146	-0.59028053	No
ATF2	activating transcription factor 2	37679	-0.018812906	-0.6634095	No
MAP3K7	mitogen-activated protein kinase kinase kinase 7	38109	-0.020746259	-0.6621948	No
TP53	tumor protein p53 (Li-Fraumeni syndrome)	38745	-0.024883565	-0.663588	Yes
MAPK9	mitogen-activated protein kinase 9	39075	-0.027651045	-0.65622413	Yes
NFATC3	nuclear factor of activated T-cells, cytoplasmic, calcineurin-dependent 3	39096	-0.027774427	-0.64148074	Yes
MAP3K9	mitogen-activated protein kinase kinase kinase 9	39528	-0.032229852	-0.63402194	Yes
MAP2K7	mitogen-activated protein kinase kinase 7	39748	-0.034743607	-0.6201694	Yes
CDC42	cell division cycle 42 (GTP binding protein, 25kDa)	40600	-0.048990607	-0.61346644	Yes
MAP3K3	mitogen-activated protein kinase kinase kinase 3	40969	-0.058069911	-0.59036005	Yes
NR2C2	nuclear receptor subfamily 2, group C, member 2	41018	-0.059616797	-0.55883396	Yes
TRAF6	TNF receptor-associated factor 6	41616	-0.080082476	-0.5290865	Yes
DUSP10	dual specificity phosphatase 10	41694	-0.08419624	-0.48478043	Yes
MAPK7	mitogen-activated protein kinase 7	41928	-0.10100361	-0.43495768	Yes
MAP3K7IP2	mitogen-activated protein kinase kinase kinase 7 interacting protein 2	42000	-0.10868074	-0.3770955	Yes
GADD45A	growth arrest and DNA-damage-inducible, alpha	42163	-0.137649789	-0.30551553	Yes
MAP3K2	mitogen-activated protein kinase kinase kinase 2	42224	-0.16140379	-0.21850796	Yes
DUSP4	dual specificity phosphatase 4	42246	-0.177573666	-0.12171865	Yes
JUN	jun oncogene	42270	-0.224415228	6.86E-04	Yes

Table V: Core enrichment genes in AKT pathway in CD4+ T cells from the LTNP group (VIR versus LTNP)

GENE SYMBOL	GENE_TITLE	RANK IN GENE LIST	RANK METRIC SCORE	RUNNING ES	CORE ENRICHMENT
YWHAH	tyrosine 3-monooxygenase/tryptophan 5-monooxygenase activation protein, eta polypeptide	2517	0.055171106	0.014855959	No
MLLT7	myeloid/lymphoid or mixed-lineage leukemia (trithorax homolog, Drosophila); translocated to, 7	4341	0.025529198	0.006160976	No
CASP9	caspase 9, apoptosis-related cysteine peptidase	5019	0.021175863	0.01869978	No
PDPK1	3-phosphoinositide dependent protein kinase-1	17883	0.002881675	-0.28162715	No
GHR	growth hormone receptor	20123	0.0012625	-0.33287773	No
AKT1	v-akt murine thymoma viral oncogene homolog 1	22755	-6.64E-04	-0.39420593	No
BAD	BCL2-antagonist of cell death	29632	-0.006159219	-0.5485204	No
CHUK	conserved helix-loop-helix ubiquitous kinase	31397	-0.007945098	-0.5795275	No
GH1	growth hormone 1	34245	-0.01177122	-0.6309892	No
FOXO3A	forkhead box O3A	37090	-0.019546116	-0.67189753	Yes
PPP2CA	protein phosphatase 2 (formerly 2A), catalytic subunit, alpha isoform	38837	-0.035149679	-0.66580087	Yes
FOXO1A	forkhead box O1A (rhabdomyosarcoma)	39474	-0.045946155	-0.6188963	Yes
NFKB1	nuclear factor of kappa light polypeptide gene enhancer in B-cells 1 (p105)	39571	-0.048017658	-0.5564278	Yes
NFKBIA	nuclear factor of kappa light polypeptide gene enhancer in B-cells inhibitor, alpha	39626	-0.049282972	-0.49126002	Yes
RELA	v-rel reticuloendotheliosis viral oncogene homolog A, nuclear factor of kappa light polypeptide gene enhancer in B-cells 3, p65 (avian)	40830	-0.081437863	-0.40991408	Yes
PIK3R1	phosphoinositide-3-kinase, regulatory subunit 1 (p85 alpha)	41392	-0.110227838	-0.27456918	Yes
PIK3CA	phosphoinositide-3-kinase, catalytic, alpha polypeptide	42199	-0.219544008	0.00236494	Yes

Table VI: Core enrichment genes in WNT pathway in CD4+ T cells from the LTNP group (VIR versus LTNP)

GENE SYMBOL	GENE_TITLE	RANK IN GENE LIST	RANK METRIC SCORE	RUNNING ES	CORE ENRICHMENT
LDLR	low density lipoprotein receptor (familial hypercholesterolemia)	122	0.238274649	0.07287744	No
FZD2	frizzled homolog 2 (Drosophila)	498	0.149843335	0.11164623	No
CCND3	cyclin D3	1600	0.080546252	0.11119268	No
PRKCD	protein kinase C, delta	1995	0.069011152	0.12380894	No
MYC	v-myc myelocytomatosis viral oncogene homolog (avian)	2163	0.06464649	0.14041139	No
PAFAH1B1	platelet-activating factor acetylhydrolase, isoform Ib, alpha subunit 45kDa	2172	0.064294294	0.16066606	No
PRKCB1	protein kinase C, beta 1	2804	0.049093019	0.16133803	No
CCND1	cyclin D1	3614	0.034201194	0.15306075	No
GSK3B	glycogen synthase kinase 3 beta	3785	0.031647481	0.15909927	No
WNT2B	wingless-type MMTV integration site family, member 2B	5328	0.019766951	0.12887901	No
WNT2	wingless-type MMTV integration site family member 2	5612	0.018584907	0.12808876	No
FRAT1	frequently rearranged in advanced T-cell lymphomas	5936	0.01743078	0.12598456	No
FZD1	frizzled homolog 1 (Drosophila)	6662	0.015474289	0.11374119	No
PRKCE	protein kinase C, epsilon	6967	0.014808014	0.111252815	No
FZD5	frizzled homolog 5 (Drosophila)	7421	0.013935647	0.10495959	No
WNT16	wingless-type MMTV integration site family, member 16	8482	0.01212365	0.08371993	No
RHOA	ras homolog gene family, member A	8579	0.011986039	0.08525848	No
FZD9	frizzled homolog 9 (Drosophila)	9331	0.01085956	0.0709322	No
WNT3	wingless-type MMTV integration site family, member 3	14302	0.005697284	-0.044917174	No
WNT5A	wingless-type MMTV integration site family, member 5A	14368	0.005646983	-0.044660393	No
FOSL1	FOS-like antigen 1	14542	0.005497712	-0.047007896	No
WNT5B	wingless-type MMTV integration site family, member 5B	15397	0.004805066	-0.065697804	No

WNT4	wingless-type MMTV integration site family, member 4	15558	0.004684093	-0.06799625	No
FZD7	frizzled homolog 7 (Drosophila)	17539	0.003165675	-0.11386464	No
PLAU	plasminogen activator, urokinase	18515	0.002426252	-0.13617553	No
FZD10	frizzled homolog 10 (Drosophila)	19151	0.001952651	-0.15058778	No
FBXW2	F-box and WD-40 domain protein 2	19460	0.001745423	-0.15732445	No
DVL2	dishevelled, dsh homolog 2 (Drosophila)	24853	-0.002242281	-0.28426296	No
PRKCG	protein kinase C, gamma	25013	-0.002361305	-0.28727633	No
WNT6	wingless-type MMTV integration site family, member 6	25976	-0.003112756	-0.30906117	No
FZD8	frizzled homolog 8 (Drosophila)	26397	-0.003451389	-0.3179069	No
WNT7B	wingless-type MMTV integration site family, member 7B	27311	-0.004175694	-0.33819368	No
MAPK10	mitogen-activated protein kinase 10	29095	-0.00564577	-0.37860966	No
WNT11	wingless-type MMTV integration site family, member 11	29640	-0.006164006	-0.38952842	No
APC	adenomatosis polyposis coli	30004	-0.006505246	-0.39605367	No
PPP2R5E	protein phosphatase 2, regulatory subunit B (B56), epsilon isoform	30399	-0.00690921	-0.40318435	No
DVL1	dishevelled, dsh homolog 1 (Drosophila)	32096	-0.008734114	-0.4405586	No
SFRP4	secreted frizzled-related protein 4	34085	-0.011486586	-0.48397055	No
PRKD1	protein kinase D1	35400	-0.014031571	-0.5106168	No
AXIN1	axin 1	36262	-0.016371315	-0.5257946	No
RAC1	ras-related C3 botulinum toxin substrate 1 (rho family, small GTP binding protein Rac1)	36826	-0.018440273	-0.53325963	No
FZD3	frizzled homolog 3 (Drosophila)	36935	-0.018931488	-0.5297967	No
MAPK9	mitogen-activated protein kinase 9	38165	-0.027195634	-0.5502448	No
FZD6	frizzled homolog 6 (Drosophila)	39098	-0.039064907	-0.55988747	Yes
DVL3	dishevelled, dsh homolog 3 (Drosophila)	39572	-0.04802281	-0.5558153	Yes
WNT10A	wingless-type MMTV integration site family, member 10A	40092	-0.059116464	-0.54930454	Yes
PRKCI	protein kinase C, iota	40140	-0.060057353	-0.53132045	Yes
PRKCZ	protein kinase C, zeta	40529	-0.070644684	-0.5180427	Yes
WNT1	wingless-type MMTV integration site family, member 1	40669	-0.075194299	-0.4974234	Yes
CCND2	cyclin D2	40753	-0.078298196	-0.47449142	Yes
TCF7	transcription factor 7 (T-cell specific, HMG-box)	41179	-0.09719231	-0.45364812	Yes
CTNNB1	catenin (cadherin-associated protein), beta 1, 88kDa	41425	-0.112471499	-0.423685	Yes
WNT10B	wingless-type MMTV integration site family, member 10B	41475	-0.115708314	-0.38805255	Yes
PRKCQ	protein kinase C, theta	41659	-0.128707841	-0.3514589	Yes
PPP2R5C	protein phosphatase 2, regulatory subunit B (B56), gamma isoform	41743	-0.136108115	-0.31014472	Yes
CSNK1E	casein kinase 1, epsilon	41827	-0.144000039	-0.26632106	Yes
PRKCH	protein kinase C, eta	42128	-0.193445891	-0.21191214	Yes
PRKCA	protein kinase C, alpha	42130	-0.193834111	-0.15030116	Yes
JUN	jun oncogene	42219	-0.231110632	-0.07889678	Yes
WNT7A	wingless-type MMTV integration site family, member 7A	42252	-0.254003167	0.001112634	Yes

Table VII: Core enrichment genes in OXPHOS pathway in CD8+ T cells from the VIR group (VIR versus LTNP)

GENE SYMBOL	GENE_TITLE	RANK IN GENE LIST	RANK METRIC SCORE	RUNNING ES	CORE ENRICHMENT
ATP6V1D	ATPase, H+ transporting, lysosomal 34kDa, V1 subunit D	25	0.422813028	0.12024352	Yes
NDUFB5	NADH dehydrogenase (ubiquinone) 1 beta subcomplex, 5, 16kDa	218	0.264094472	0.1911736	Yes
PPA2	pyrophosphatase (inorganic) 2	313	0.238639355	0.25714883	Yes
NDUFA8	NADH dehydrogenase (ubiquinone) 1 alpha subcomplex, 8, 19kDa	607	0.18775773	0.30387172	Yes
UQCRC1	ubiquinol-cytochrome c reductase core protein I	647	0.182142124	0.3550027	Yes
COX6B1	cytochrome c oxidase subunit Vib polypeptide 1 (ubiquitous)	1073	0.143539995	0.3859638	Yes
COX7B	cytochrome c oxidase subunit VIIb	1251	0.132460684	0.41962945	Yes
COX5A	cytochrome c oxidase subunit Va	1427	0.122701913	0.45055354	Yes
COX10	COX10 homolog, cytochrome c oxidase assembly protein, heme A: farnesyltransferase (ye	1898	0.098998234	0.4677198	Yes
NDUFB6	NADH dehydrogenase (ubiquinone) 1 beta subcomplex, 6, 17kDa	2100	0.089572906	0.48856044	Yes
NDUFS1	NADH dehydrogenase (ubiquinone) Fe-S protein 1, 75kDa (NADH-coenzyme Q reductas	2406	0.077389516	0.50345725	Yes
NDUFB2	NADH dehydrogenase (ubiquinone) 1 beta subcomplex, 2, 8kDa	2474	0.075391836	0.5234173	Yes
NDUFB7	NADH dehydrogenase (ubiquinone) 1 beta subcomplex, 7, 18kDa	2614	0.071086399	0.54044247	Yes
ATP6V1C1	ATPase, H+ transporting, lysosomal 42kDa, V1 subunit C1	2618	0.071029767	0.560671	Yes
NDUFA11	NADH dehydrogenase (ubiquinone) 1 alpha subcomplex, 11, 14.7kDa	2645	0.070307441	0.5801485	Yes
ATP5E	ATP synthase, H+ transporting, mitochondrial F1 complex, epsilon subunit	2929	0.06189033	0.59113663	Yes
ATP6V1B1	ATPase, H+ transporting, lysosomal 56/58kDa, V1 subunit B1 (Renal tubular acidosis with	3078	0.057863083	0.60416967	Yes
UQCRB	ubiquinol-cytochrome c reductase binding protein	3182	0.055413418	0.6175679	Yes
ATP6V1A	ATPase, H+ transporting, lysosomal 70kDa, V1 subunit A	3353	0.051970046	0.6283959	Yes
COX6A1	cytochrome c oxidase subunit VIa polypeptide 1	3475	0.049450766	0.63966393	Yes
UQCRH	ubiquinol-cytochrome c reductase hinge protein	3660	0.04561583	0.6483446	Yes
ATP7A	ATPase, Cu++ transporting, alpha polypeptide (Menkes syndrome)	3690	0.045052134	0.6605335	Yes

Rank in gene list[a]: the individual rank of a gene in relation to the phenotype distinction among all genes on the microarray

Rank metric score[b]: metric score used for gene rank

Running ES[c]: calculation of the enrichment score which reflects the extend of the overrepresentation of a gene set in a phenotype group

Core enrichment[d]: an indication whether a particular gene within the gene set contributes to the overrepresentation of this gene set in a phenotype group
