# Supplementary figures and images for: Genome-wide analysis of primary CD4+ and CD8+ T cell transcriptomes shows evidence for a network of enriched pathways associated with HIV disease
Source: Retrovirology. 2011 Mar 16;8:18. doi: 10.1186/1742-4690-8-18 (PMC3068086; doi:10.1186/1742-4690-8-18)

## COMPLEMENT AND COAGULATION CASCADES

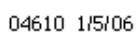

Supplement: Additional file 3 — Core enrichment genes (highlighted in red) in the complement and coagulation cascade. Figure of complement and coagulation cascade pathway with highlighted genes. [file 1742-4690-8-18-S3.PDF]
