# Supplementary material for: Genome-wide analysis of primary CD4+ and CD8+ T cell transcriptomes shows evidence for a network of enriched pathways associated with HIV disease
Source: Retrovirology. 2011 Mar 16;8:18. doi: 10.1186/1742-4690-8-18 (PMC3068086; doi:10.1186/1742-4690-8-18)
Supplement: Additional file 4 — Top ranked gene sets enriched in the LTNP group. List of top ranked gene sets enriched in the LTNP group. [file 1742-4690-8-18-S4.PDF]

Supplementary file 4: Top ranked gene sets enriched in the LTNP group

Table 1: BDL versus LTNP in CD8+ T cells

GENE SET NAME	GENE NUM	ES	NES	NOM p-val	FDR
INFLAMPATHWAY	29	-0.76645	-1.9618	0	0.00471
HSA05010_ALZHEIMERS_DISEASE	27	-0.71716	-1.82227	0	0.06424
**NGFPATHWAY**	19	-0.738	-1.74851	0.002066116	0.08662
HYPERTROPHY_MODEL	17	-0.7467	-1.73308	0.003992016	0.08754
TH1TH2PATHWAY	17	-0.7566	-1.75694	0	0.09252
ATMPATHWAY	19	-0.73821	-1.76417	0.005882353	0.10845
STATIN_PATHWAY_PHARMGKB	17	-0.72772	-1.69466	0.007532957	0.12674
NKTPATHWAY	28	-0.64083	-1.65656	0.014492754	0.17052
HSA04012_ERBB_SIGNALING_PATHWAY	87	-0.5131	-1.63434	0.004282655	0.17645
ARAPPATHWAY	20	-0.68161	-1.63451	0.00610998	0.19552
CYTOKINEPATHWAY	20	-0.65598	-1.59786	0.013888889	0.19744
IL1RPATHWAY	32	-0.60176	-1.59794	0.014492754	0.2139
MRNA_PROCESSING_REACTOME	104	-0.49741	-1.6049	0.002159827	0.2153
IL12PATHWAY	20	-0.63957	-1.55775	0.033826638	0.21947
HSA04120_UBIQUITIN_MEDIATED_PROTEOLYSIS	37	-0.57597	-1.57187	0.018367346	0.22027
GLYCOSPHINGOLIPID_METABOLISM	21	-0.63598	-1.53416	0.039045554	0.22035
HSA04664_FC_EPSILON_RI_SIGNALING_PATHWAY	75	-0.50732	-1.57794	0.00443459	0.22407
P53HYPOXIAPATHWAY	19	-0.66235	-1.55941	0.033203125	0.22947
RELAPATHWAY	16	-0.67587	-1.5344	0.041152265	0.2309
**IGF1PATHWAY**	20	-0.63837	-1.51906	0.03773585	0.23619
HSA05221_ACUTE_MYELOID_LEUKEMIA	53	-0.52503	-1.53497	0.014227643	0.24199
**INSULINPATHWAY**	21	-0.63372	-1.51013	0.043392505	0.24245
NUCLEAR_RECEPTORS	38	-0.56434	-1.53877	0.022540983	0.24806
NFKBPATHWAY	23	-0.60601	-1.4787	0.055888224	0.26496
SA_TRKA_RECEPTOR	16	-0.62926	-1.44416	0.072186835	0.26974
HSA04920_ADIPOCYTOKINE_SIGNALING_PATHWAY	72	-0.48089	-1.49099	0.01039501	0.27138
TIDPATHWAY	18	-0.63302	-1.47893	0.048732944	0.2746
**NTHIPATHWAY**	22	-0.59567	-1.45015	0.06822612	0.27564
TNFR1PATHWAY	28	-0.55032	-1.44449	0.06097561	0.27742
IL6PATHWAY	21	-0.59574	-1.46613	0.053169735	0.28014
**ST_JNK_MAPK_PATHWAY**	40	-0.51962	-1.46026	0.030368764	0.28287
EPOPATHWAY	19	-0.62741	-1.48002	0.048582997	0.28306
CIRCADIAN_EXERCISE	40	-0.51345	-1.45029	0.04517454	0.28429
PTDINSPATHWAY	22	-0.59834	-1.45242	0.054	0.28967
**AKTPATHWAY**	17	-0.61092	-1.42608	0.06736842	0.29741

Table 2: VIR versus LTNP in CD4+ T cells

GENE SET NAME	GENE NUM	ES	NES	NOM p-val	FDR
HSA03010_RIBOSOME	83	-0.69324	-1.9468	0	8.16E-04
RIBOSOMAL_PROTEINS	86	-0.66951	-1.88056	0	0.00629
CIRCADIAN_EXERCISE	40	-0.69685	-1.74798	0.004366812	0.05276
**ST_JNK_MAPK_PATHWAY**	40	-0.7008	-1.76293	0	0.05556
CALCINEURIN_NF_AT_SIGNALING	92	-0.56628	-1.62043	0	0.19497
ATMPATHWAY	19	-0.76347	-1.64638	0.010660981	0.19616
**NGFPATHWAY**	19	-0.73915	-1.57899	0.024590164	0.21522
TOB1PATHWAY	16	-0.71136	-1.51045	0.04904051	0.21967
PAR1PATHWAY	20	-0.68169	-1.50362	0.042682927	0.2224
CCR5PATHWAY	18	-0.69329	-1.49749	0.03846154	0.22394
CARDIACEGFPATHWAY	17	-0.77173	-1.62068	0.010224949	0.22662
**AKTPATHWAY**	17	-0.71319	-1.5122	0.02736842	0.22905
TCRPATHWAY	43	-0.63324	-1.5831	0.008316008	0.23072
MRNA_PROCESSING_REACTOME	104	-0.55311	-1.59375	0.004694836	0.23146
PITX2PATHWAY	16	-0.70906	-1.48227	0.0390625	0.23452
HSA04660_T_CELL_RECEPTOR_SIGNALING_PATHW/	93	-0.53715	-1.5437	0.006864989	0.23563
**WNT_SIGNALING**	60	-0.57231	-1.51507	0.012903226	0.23656
CYTOKINEPATHWAY	20	-0.69849	-1.53501	0.025742574	0.23771
**NTHIPATHWAY**	22	-0.66528	-1.48527	0.030927835	0.23939
GPCRPATHWAY	35	-0.62038	-1.51648	0.038934425	0.24768
INFLAMPATHWAY	29	-0.65216	-1.52258	0.033464566	0.24884

Table 3: VIR versus LTNP in CD8+ T cells

GENE SET NAME	GENE NUM	ES	NES	NOM p-val	FDR
HSA05221_ACUTE_MYELOID_LEUKEMIA	53	-0.60018	-1.57069	0.013761468	0.261
NUCLEAR_RECEPTORS	38	-0.68855	-1.71438	0.002169197	0.261
HSA05217_BASAL_CELL_CARCINOMA	56	-0.60877	-1.57821	0.008791209	0.274
**IGF1PATHWAY**	20	-0.73528	-1.59017	0.015217391	0.279
IL1RPATHWAY	32	-0.66333	-1.60191	0.011061947	0.287
**ST_JNK_MAPK_PATHWAY**	40	-0.61963	-1.53734	0.018181818	0.298
**INSULINPATHWAY**	21	-0.7213	-1.61197	0.007561437	0.318
CIRCADIAN_EXERCISE	40	-0.62869	-1.53988	0.01594533	0.323
HSA03010_RIBOSOME	83	-0.57423	-1.62989	0.002212389	0.34
CARDIACEGFPATHWAY	17	-0.65908	-1.39979	0.07805907	0.346
**NTHIPATHWAY**	22	-0.64909	-1.43699	0.049462367	0.349

ES: enrichment score; NES: normalized enrichment score; NOM p-val: nominal p value; FDR: false discovery rate.

Gene sets in bold are significantly enriched in the LTNP group (FDR<0.1) in the comparison of BDL versus LTNP in CD4+ T cells.
